# Supplementary material for: Multi-state design of flexible proteins predicts sequences optimal for conformational change
Source: PLoS Comput Biol. 2020 Feb 7;16(2):e1007339. doi: 10.1371/journal.pcbi.1007339 (PMC7032724; doi:10.1371/journal.pcbi.1007339)
Supplement: S1 Table — The chain and residue numbers for each PDB along each row were designed separately, except for proteins with multiple chains included in design, such as influenza virus HA2 and RSV F, where a single chain and corresponding residue numbers are included on an individual line. A grey line that marks the end of all chain and residue numbers included for one PDB. The notation ([]) denotes chain breaks within the sequence. The (*) indicates that gaps in the alignment where there was incomplete density in the crystal structure. For input PDB models, including 1OK8, 3C5X and 3C6E, the sequence from 3J27 was threaded onto the missing densities in structures 1OK8, 3C5X, and 3C6E so that there were no gaps. A detailed description of the preparation of input models for design is included in the S1 Appendix. (DOCX) [file pcbi.1007339.s001.docx]

S1 Table. Structures and their residues considered for design.

| Protein | PDBs included in dataset | Designed chains within each PDB | Designed residues within each PDB |
| --- | --- | --- | --- |
| 5′-nucleotidase | 1HPU | A | 1 – 523 |
| (5′-NT) | 1OI8 | A | 1 – 523 |
|  | 1OID | A | 1 – 523 |
|  | 1OID | B | 1 – 523 |
|  | 4WWL | A | 1 – 523 |
| Adenylate kinase | 1AKE | A | 1 – 128 |
|  | 4AKE | A | 1 – 128 |
| CagL | 3ZCJ | A | 61 – 229 |
|  | 4CII | A | 61 – 229 |
|  | 4YVM | A | 61 – 229 |
| Calmodulin | 1A29 | A | 4 – 143 |
|  | 1CFC | A | 6 – 145 |
|  | 1CFD | A | 6 – 145 |
|  | 1CFF | A | 6 – 145 |
|  | 1CKK | A | 6 – 145 |
|  | 1CLL | A | 3 – 142 |
|  | 1CM1 | A | 3 – 142 |
|  | 1CM4 | A | 3 – 142 |
|  | 1G4Y | R | 87 – 226 |
|  | 1LIN | A | 4 – 143 |
|  | 1MUX | A | 6 – 145 |
|  | 1NIW | A | 4 – 143 |
|  | 1NWD | A | 6 – 145 |
|  | 2F2P | A | 5 – 144 |
|  | 2N8J | A | 6 – 145 |
|  | 2WEL | D | 309 – 448 |
|  | 3EWT | A | 6 – 145 |
|  | 3EWV | A | 3 – 142 |
|  | 4DJC | A | 9 – 148 |
|  | 4HEX | B | 140 – 279 |
| Dengue virus envelope | 1OAN | A | 1 – 394 |
| (E) protein | 1OK8* | A | 1 – 394 |
| monomer | 3C5X* | A | 1 – 394 |
|  | 3C6E* | A | 1 – 394 |
|  | 3J27 | A | 1 – 394 |
|  | 3J2P | A | 1 – 394 |
| Influenza hemagglutinin stem (HA2) trimer | 1HTM | B | 13 – 92; 102 – 119 126 – 248 |
|  |  | D | 257 – 379 |
|  |  | F |  |
|  | 1QU1 | A | 47 – 126, 136 – 153 |
|  |  | B | 40 – 162 |
|  |  | C | 40 – 162 |
|  | 2HMG | B | 47 – 126, 136 – 153 |
|  |  | D | 40 – 162 |
|  |  | F | 40 – 162 |
|  | 3EYM | B | 47 – 126, 136 – 153 |
|  |  | D | 40 – 162 |
|  |  | F | 40 – 162 |
| GroEL subunit | 1AON | A | 2 – 524 |
|  | 1AON | N | 2 – 524 |
|  | 2C7E | A | 2 – 524 |
|  | 3WVL | H | 2 – 524 |
|  | 4AB3 | A | 2 – 524 |
|  | 4KI8 | A | 2 – 524 |
| Respiratory syncytial virus | 3RKI | A | 27 - 97, 150 - 506; 27 - 97, 151 - 506; 27 - 97, 155 - 505 |
|  |  | B |  |
|  |  | C |  |
| fusion (RSV F) protein trimer | 3RRR | A | 27 – 97 |
|  |  | B | 150 – 506 |
|  |  | C | 27 – 97 |
|  |  | D | 151 – 506 |
|  |  | E | 27 – 97 |
|  |  | F | 155 – 505 |
|  | 4MMS | A | 27 – 97 |
|  |  | B | 150 – 506 |
|  |  | C | 27 – 97 |
|  |  | D | 151 – 506 |
|  |  | E | 27 – 97 |
|  |  | F | 155 - 505 |
|  | 4ZYP | A | 27 – 97, 150 – 506 27 – 97, 151 – 506 27 – 97, 155 – 505 |
|  |  | B |  |
|  |  | C |  |
| Protein | Native sequence | | |
| 5′-NT | YEQDKTYKITVLHTNDHHGHFWRNEYGEYGLAAQKTLVDGIRKEVAAEGGSVLLLSGGDINTGVPESDLQDAEPDFRGMNLVGYDAMAIGNHEFDNPLTVLRQQEKWAKFPLLSANIYQKSTGERLFKPWALFKRQDLKIAVIGLTTDDTAKIGNPEYFTDIEFRKPADEAKLVIQELQQTEKPDIIIAATHMGHYDNGEHGCNAPGDVEMARALPAGSLAMIVGGHSQDPVCMAAENKKQVDYVPGTPCKPDQQNGIWIVQAHEWGKYVGRADFEFRNGEMKMVNYQLIPVNLKKKVTWEDGKSERVLYTPEIAENQQMISLLSPFQNKGKAQLEVKIGETNGRLEGDRDKVRFVQTNMGRLILAAQMDRTGADFAVMSGGGIRDSIEAGDISYKNVLKVQPFGNVVVYADMTGKEVIDYLTAVAQMKPDSGAYPQFANVSFVAKDGKLNDLKIKGEPVDPAKTYRMATLNFNATGGDGYPRLDNKCGYVNT | | |
| Adenylate kinase | MRIILLGAPGAGKGTQAQFIMEKYGIPQISTGDMLRAAVKSGSELGKQAKDIMDAGKLVTDELVIALVKERIAQEDCRNGFLLDGFPRTIPQADAMKEAGINVDYVLEFDVPDELIVDRIVGRRVHAPSGRVYHVKFNPPKVEGKDDVTGEELTTRKDDQEETVRKRLVEYHQMTAPLIGYYSKEAEAGNTKYAKVDGTKPVAEVRADLEKILG | | |
| CagL | GEEDALNIKKAAIALRGDLALLKANFEANELFFISEDVIFKTYMSSPELLLTYMKINPLDQNTAEQQCGISDKVLVLYCEGKLKIEQEKQNIRERLETSLKAYQSNIGGTASLITASQTLVESLKNKNFIKGIRKLMLAHNKVFLNYLEELDALERSLEQSKRQYLQER | | |
| Calmodulin | EEQIAEFKEAFSLFDKDGDGTITTKELGTVMRSLGQNPTEAELQDMINEVDADGNGTIDFPEFLTMMARKMKDTDSEEEIREAFRVFDKDGNGYISAAELRHVMTNLGEKLTDEEVDEMIREADIDGDGQVNYEEFVQMM | | |
| Dengue virus E monomer | MRCIGISNRDFVEGVSGGSWVDIVLEHGSCVTTMAKNKPTLDFELIETEAKQPATLRKYCIEAKLTNTTTDSRCPTQGEPSLNEEQDKRFVCKHSMVDRGWGNGCGLFGKGGIVTCAMFTCKKNMKGKVVQPENLEYTIVITPHSGEEHAVGNDTGKHGKEIKITPQSSITEAELTGYGTVTMECSPRTGLDFNEMVLLQMENKAWLVHRQWFLDLPLPWLPGADTQGSNWIQKETLVTFKNPHAKKQDVVVLGSQEGAMHTALTGATEIQMSSGNLLFTGHLKCRLRMDKLQLKGMSYSMCTGKFKVVKEIAETQHGTIVIRVQYEGDGSPCKIPFEIMDLEKRHVLGRLITVNPIVTEKDSPVNIEAEPPFGDSYIIIGVEPGQLKLNWFKK | | |
| Influenza virus HA2 trimer | QINGKLNRVIEKTNEKFHQIEKEFSEVEGRIQDLEKYVEDTKIDLWSYNAELLVALENQHTIDLTDSEMNKLFEKTRRQLGSFKIYHKCDNACIESIR[] | | |
|  | STQAAIDQINGKLNRVIEKTNEKFHQIEKEFSEVEGRIQDLEKYVEDTKIDLWSYNAELLVALENQHTIDLTDSEMNKLFEKTRRQLRENAEEMGNGSFKIYHKCDNACIESIRNGTYDHDVY[] | | |
|  | STQAAIDQINGKLNRVIEKTNEKFHQIEKEFSEVEGRIQDLEKYVEDTKIDLWSYNAELLVALENQHTIDLTDSEMNKLFEKTRRQLRENAEEMGNGSFKIYHKCDNACIESIRNGTYDHDVY[] | | |
| GroEL subunit | AAKDVKFGNDARVKMLRGVNVLADAVKVTLGPKGRNVVLDKSFGAPTITKDGVSVAREIELEDKFENMGAQMVKEVASKANAAAGDGTTTATVLAQAIITEGLKAVAAGMNPMDLKRGIDKAVTAAVEELKALSVPCSDSKAIAQVGTISANSDETVGKLIAEAMDKVGKEGVITVEDGTGLQDELDVVEGMQFDAGYLSPYFINKPETGAVELESPFILLADKKISNIREMLPVLEAVAKAGKPLLIIAEDVEGEALATLVVNTMRGIVKVAAVKAPGFGDRRKAMLQDIATLTGGTVISEEIGMELEKATLEDLGQAKRVVINKDTTTIIDGVGEEAAIQGRVAQIRQQIEEATSDYDREKLQERVAKLAGGVAVIKVGAATEVEMKEKKARVEDALHATRAAVEEGVVAGGGVALIRVASKLADLRGQNEDQNVGIKVALRAMEAPLRQIVLNCGEEPSVVANTVKGGDGNYGYNAATEEYGNMIDMGILDPTKVTRSALQYAASVAGLMITTECMVTDL | | |
| RSV F trimer | NITEEFYQSTCSAVSKGYLSALRTGWYTSVITIELSNIKENKCNGTDAKVKLIKQELDKYKNAVTELQLLM [] | | |
|  | SGVAVSKVLHLEGEVNKIKSALLSTNKAVVSLSNGVSVLTFKVLDLKNYIDKQLLPILNKQSCSISNIETVIEFQQKNNRLLEITREFSVNAGVTTPVSTYMLTNSELLSLINDMPITNDQKKLMSNNVQIVRQQSYSIMSIIKEEVLAYVVQLPLYGVIDTPCWKLHTSPLCTTNTKEGSNICLTRTDRGWYCDNAGSVSFFPQAETCKVQSNRVFCDTMNSLTLPSEVNLCNVDIFNPKYDCKIMTSKTDVSSSVITSLGAIVSCYGKTKCTASNKNRGIIKTFSNGCDYVSNKGVDTVSVGNTLYYVNKQEGKSLYVKGEPIINFYDPLVFPSDEFDASISQVNEKINQSLAFI [] | | |
|  | NITEEFYQSTCSAVSKGYLSALRTGWYTSVITIELSNIKENKCNGTDAKVKLIKQELDKYKNAVTELQLLM [] | | |
|  | GVAVSKVLHLEGEVNKIKSALLSTNKAVVSLSNGVSVLTFKVLDLKNYIDKQLLPILNKQSCSISNIETVIEFQQKNNRLLEITREFSVNAGVTTPVSTYMLTNSELLSLINDMPITNDQKKLMSNNVQIVRQQSYSIMSIIKEEVLAYVVQLPLYGVIDTPCWKLHTSPLCTTNTKEGSNICLTRTDRGWYCDNAGSVSFFPQAETCKVQSNRVFCDTMNSLTLPSEVNLCNVDIFNPKYDCKIMTSKTDVSSSVITSLGAIVSCYGKTKCTASNKNRGIIKTFSNGCDYVSNKGVDTVSVGNTLYYVNKQEGKSLYVKGEPIINFYDPLVFPSDEFDASISQVNEKINQSLAFI [] | | |
|  | NITEEFYQSTCSAVSKGYLSALRTGWYTSVITIELSNIKENKCNGTDAKVKLIKQELDKYKNAVTELQLLM [] | | |
|  | SKVLHLEGEVNKIKSALLSTNKAVVSLSNGVSVLTFKVLDLKNYIDKQLLPILNKQSCSISNIETVIEFQQKNNRLLEITREFSVNAGVTTPVSTYMLTNSELLSLINDMPITNDQKKLMSNNVQIVRQQSYSIMSIIKEEVLAYVVQLPLYGVIDTPCWKLHTSPLCTTNTKEGSNICLTRTDRGWYCDNAGSVSFFPQAETCKVQSNRVFCDTMNSLTLPSEVNLCNVDIFNPKYDCKIMTSKTDVSSSVITSLGAIVSCYGKTKCTASNKNRGIIKTFSNGCDYVSNKGVDTVSVGNTLYYVNKQEGKSLYVKGEPIINFYDPLVFPSDEFDASISQVNEKINQSLAF [] | | |

The chain and residue numbers for each PDB along each row were designed separately, except for proteins with multiple chains included in design, such as influenza virus HA2 and RSV F, where a single chain and corresponding residue numbers are included on an individual line, where a grey line marks the end of each all chain and residue numbers included for one PDB. [] denotes chain breaks within the sequence. The (*) indicates that gaps in the alignment where there was incomplete density in the crystal structure, which include at most three missing residues for 1OK8 and two residues for 3C5X and 3C6E. For these input models, the sequence from 3J2P was threaded onto the missing densities in structures 1OK8, 3C5X, and 3C6E so that there were no gaps.
