# Supplementary material for: Multi-state design of flexible proteins predicts sequences optimal for conformational change
Source: PLoS Comput Biol. 2020 Feb 7;16(2):e1007339. doi: 10.1371/journal.pcbi.1007339 (PMC7032724; doi:10.1371/journal.pcbi.1007339)
Supplement: S1 Appendix — The following document includes a detailed description of model preparation, protein design, and analysis methods used in this manuscript, including the software versions and command line options. Command line options are written in monospace. The ‘\\’ symbol when included in command line options indicates a wrapped single line. Scripts requiring either a Python or R environment are indicated. (PDF) [file pcbi.1007339.s002.pdf]

SI Appendix: Protocol capture

Introduction

The following protocol capture describes how to run RECON multi-state design and single-state design and the analyses performed that were discussed within the manuscript. For simplicity, we use only one of the eight protein ensembles included in the benchmark, dengue virus envelope (DV E) protein, as a case example to run all scripts and analyses.

All native structures, relaxed structures, Rosetta scripts, and other analysis scripts used in this benchmark can be downloaded from https://github.com/mfsauer/RECON_flexible_sequences.

Dependencies

All Rosetta commands for this publication were run with version 6b77f113505c4687d084d54890b1027ff308330d, from March 2016. Note that all analysis scripts will only function properly if they are in the correct directory as provided.

Several scripts used in this protocol require Python, either Python 2.7 or Python 3.7, and the required version is noted for each script. Additionally, several scripts require the Biopython package (https://github.com/biopython/biopython.github.io/). It is recommended that the user have both versions of Python and the Biopython package installed prior to using this protocol.

To generate sequences profiles, the user can install WebLogo locally either using `pip` or downloaded manually from https://github.com/WebLogo/weblogo. Multiple sequence alignments require Clustal Omega —for generating alignments of input structures or designs, the user may use the online version found at https://www.ebi.ac.uk/Tools/msa/clustalo/ or download a local version found at http://www.clustal.org/omega/#Download.

For plotting, it is recommended that the user have R with the following packages installed into their library: ggplot2, cowplot, ape, broom, dendextend, dendsort, ggdendro, ggpubr, ggrepel, ggridges, ggsignif, Hmisc, Kendall, latex2exp, plotly, reshape2, stats, and treeio.

Structure Preparation

All structures (1OAN, 1OK8, 3C5X, 3C6E, 3J27, and 3J2P) were downloaded from the Protein DataBank (PDB; www.rcsb.org) and manually processed in PyMol to remove all waters and non-protein atoms. The FASTA sequence of each chain was generated using

```
python2.7 get_fasta_from_pdb.py 1oan A > 1oan_A.fa
```

DV E protein is a single chain, but for multi-chain proteins the individual `.fa` files were concatenated to form a single `.fa` file of the whole protein. Using the aligned sequences, any residues not aligned at either the N- or C-termini were removed from the original PDB file using PyMol and saved as the native PDB for relaxation and design. For all other protein ensembles, any gaps in sequence alignment were excluded from design. However, for DV E structures 1OK8, 3C5X, and 3C6E, missing densities were replaced (so that the entire E monomer could be designed) using the Rosetta Partial Thread application as follows:

A grishin file was generated for each of the three DV E structures to define where the missing densities were located. Again, for clarity, we describe only the partial thread procedure for 3C6E, but the same protocol was applied in all cases. Below is the ROSETTA partial thread application script along with the needed files. The sequence from 1OAN was used as the threaded sequence to fill any gaps in sequence for Any text denoted between two ---- indicates a separate file containing the contents between the two lines with the file name indicated on the top line. Any \\ notation indicates that the command line continues with no return.

```
/path/to/rosetta/main/source/bin/partial_thread.default.linuxgccrelease \\
-in:file:fasta 1oan_A.fasta -in:file:alignment 3c6e.grishin \\
-in:file:template 3c6e.pdb

----------------------------------3c6e.grishin----------------------------------
## 1oanA 3c6eA
# hhsearch
 scores_from_program 0 1.00
 0 MRCIGISNRDFVEGVSGGSWVDIVLEHGSCVTTMAKNKPTLDFELIKTEAKQPATLRKYCIEAKLTNTTTESRC
PTQGEPTLNEEQDKRFVCKHSMVDRGWGNGCGLFGKGGIVTCAMFTCKKNMEGKIVQPENLEYTVVITPHSGEEHA
VGNDTGKHGKEVKITPQSSITEAELTGYGTVTMECSPRTGLDFNEMVLLQMKDKAWLVHRQWFLDLPLPWLPGADT
QGSNWIQKETLVTFKNPHAKKQDVVVLGSQEGAMHTALTGATEIQMSSGNLLFTGHLKCRLRMDKLQLKGMSYSMC
TGKFKVVKEIAETQHGTIVIRVQYEGDGSPCKIPFEIMDLEKRHVLGRLITVNPIVTEKDSPVNIEAEPPFGDSYI
IIGVEPGQLKLNWFKK
 0 MRCIGMSNRDFVEGVSGGSWVDIVLEHGSCVTTMAKNKPTLDFELIKTEAKQPATLRKYCIEAKLTNTTTESRC
PTQGEPSLNEEQDKRFVCKHSMVDRGWGNGCGLFGKGGIVTCAMFRCKKNMEGKVVQPENLEYTIVITPHSGEEHA
————GKHGKEIKITPQSSITEAELTGYGTVTMECSPRT-LDFEMVLLQMENKAWLVHRQWFLDLPLPWLPGADT
QGSNWIQKETLVTFKNPHAKKQDVVVLGSQEGAMHTALTGATEIQMSSGNLLFTGHLKCRLRMDKLQLKGMSYSMC
TGKFKVVKEIAETQHGTIVIRVQY-GDGSPCKIPFEIMDLEKRHVLGRLITVNPIVTEKDSPVNIEAEPPFGDSYI
IIGVEPGQLKLNWFKK
--
--------------------------------------------------------------------------------

# After running rename output model
mv 3c6e.pdb.pdb 3c6e_threaded.pdb
```

The partial thread application maps the missing sequence to the template structure, `3c6e.pdb`, containing the missing densities. From the threaded model, the missing backbone and side chains are rebuilt using the ROSETTA application RosettaCM hybridize

```
/path/to/rosetta/main/source/bin/rosetta_scripts.default.linuxgccrelease \\
@rosetta_cm.options -s 3c6e_threaded.pdb

------------------------------rosetta_cm.options------------------------------
-database /path/to/rosetta/main/database
-parser:protocol hybridize.xml
-default_max_cycles 200
-dualspace
--------------------------------------------------------------------------------

--------------------------------hybridize.xml-------------------------------
<ROSETTASCRIPTS>
<SCOREFXNS>
    <stage1 weights=score3 symmetric=0>
        <Reweight scoretype=atom_pair_constraint weight=0.5/>
```

```
    </stage1>
    <stage2 weights=score4_smooth_cart symmetric=0>
        <Reweight scoretype=atom_pair_constraint weight=0.5/>
    </stage2>
    <fullatom weights=talaris2013_cart symmetric=0>
        <Reweight scoretype=atom_pair_constraint weight=0.5/>
    </fullatom>
</SCOREFXNS>
<MOVERS>
<Hybridize name=hybridize stage1_scorefxn=stage1 \\
stage2_scorefxn=stage2 fa_scorefxn=fullatom batch=1 \\
stage1_increase_cycles=1.0 stage2_increase_cycles=1.0>
    <Template pdb="3c6e_threadedl.pdb" cst_file="AUTO" weight=1.000 />
</Hybridize>
</MOVERS>
<PROTOCOLS>
    <Add mover=hybridize/>
</PROTOCOLS>
<OUTPUT scorefxn=talaris2013 />
</ROSETTASCRIPTS>
------------------------------------------------------------------------------
```

Refinement of input structures

All native structures were subject to a constrained FastRelax prior to design:

```
/path/to/rosetta/main/source/bin/rosetta_scripts.default.linuxgccrelease \\
@relax.flags -s 3c6e_rebuilt.pdb -scorefile 3c6e_relaxed.fasc

-----------------relax.flags-----------------
-database /path/to/rosetta/main/database/
-linmem_ig 10
-in:file:fullatom
-in:detect_disulf false
-relax:fast
-relax:constrain_relax_to_start_coords
-out:file:fullatom
-out:suffix _relax
-use_input_sc
-nstruct 100
---------------------------------------------
```

The lowest scoring model ranked by total score was chosen as the relaxed model, labeled `*_relaxed.pdb`.

Design of ensembles

For each ensemble, a resfile was created to specifiy which residues were to be considered for design. In either RECON multi-specificity or single-state design, the same resfile was used. For design of the DV E ensemble design, residues 1-394 were considered for design, and all six PDB files contained Chain A of the E monomer, which were used for design. Supplementary Table 1 lists the number, chain, and native residue considered for design for each PDB file —the matched number and chain correspond to the first and second column of each resfile. If the chain or residue numbering differed between PDB files of the same protein ensemble,

separate resfiles were created for each protein. Although not relevant in the DV E example, it is paramount that the same number of positions are listed in each resfile and that each position listed in the same order for all conformations/PDB files, should there be more than one resfile needed per ensemble. This is because each matching/equivalent position between resfiles will be modeled as a matching side chain state within an ensemble. Below is an example of the start and end of a resfile in this format:

```
-------------------denvE.resfile -------------------
NATRO
start
1 A ALLAA
2 A ALLAA
3 A ALLAA
...             # Continue for residues 4-393
394 A ALLAA
-----------------------------------------------------
```

RECON multi-state design

As mentioned in the manuscript, all aligned positions within each conformation were considered for design in protein ensembles as long as the the protein ensemble contained at least two conformations/PDB files that had a root mean square distance of 5 Å and at least 120 aligned positions of the same sequence. For RECON multi-state design of the DV E ensemble, the following scripts were used:

```
mkdir designs/              # Create directory for output models

mpiexec -n 6 \\
/path/to/rosetta/main/source/bin/rosetta_scripts.mpi.linuxgccrelease \\
@msd.options -l models.list -scorefile denvE-msd.fasc
```

```
-------------------------models.list -------------------------
1oan_diff.pdb
1ok8_diff.pdb
3c5x_diff.pdb
3c6e_diff.pdb
3j27.pdb
3j2p_diff.pdb
---------------------------------------------------------------
```

```
-------------------------msd.options-------------------------
-database /path/to/rosetta/main/database
-in:file:fullatom
-in:detect_disulf false
-mute protocols.simple_moves.GenericMonteCarloMover
-parser:protocol msd.xml
-run:msd_job_dist
-use_input_sc
-linmem_ig 50
-out:file:fullatom
-out:pdb_gz
-out:suffix _msd_
```

```
-out:path:pdb designs/
-nstruct 1
```

```
----------------------------------msd.xml----------------------------------
<ROSETTASCRIPTS>
    <SCOREFXNS>
        <tal weights=talaris2013.wts >
            <Reweight scoretype=res_type_constraint weight=1.0 />
        </tal>
    </SCOREFXNS>
    <TASKOPERATIONS>
        <InitializeFromCommandline name=ifcl />
        <RestrictToRepacking name=rtr />
    </TASKOPERATIONS>
    <MOVERS>
        <PackRotamersMover name=design scorefxn=tal task_operations=ifcl />
        <MSDMover name=msd1 design_mover=design constraint_weight=0.5 \\
        resfiles=denvE.resfile debug=1 />
        <MSDMover name=msd2 design_mover=design constraint_weight=1.0 \\
        resfiles=denvE.resfile debug=1 />
        <MSDMover name=msd3 design_mover=design constraint_weight=1.5 \\
        resfiles=denvE.resfile debug=1 />
        <MSDMover name=msd4 design_mover=design constraint_weight=2.0 \\
        resfiles=denvE.resfile debug=1 />
        <FindConsensusSequence name=finish scorefxn=tal \\
        resfiles=denvE.resfile debug=1 task_operations=ifcl \\
        repack_one_res=1 />
        <FastRelax name=relax scorefxn=talaris2013 \\
        task_operations=ifcl,rtr repeats=1 />
    </MOVERS>
    <FILTERS>
        <FitnessFilter name=fitness output_to_scorefile=1 />
    </FILTERS>
    <APPLY_TO_POSE>
    </APPLY_TO_POSE>
    <PROTOCOLS>
        <Add mover=msd1 />
        <Add mover=msd2 />
        <Add mover=msd3 />
        <Add mover=msd4 />
        <Add mover=finish />
        <Add filter=fitness />
        <Add mover=relax />
      </PROTOCOLS>
</ROSETTASCRIPTS>
```

This protocol will run in parallel over 6 processors, one for each state. For the benchmark, this protocol was run 100 times to generate 100 designed ensembles of DV E. The protocol will run four rounds of

multi-state design followed by repacking only, not a subsequent minimization as described previously, to avoid over-optimization to the Rosetta energy score function during design and to conserve the peptide backbone geometry of the original relaxed state. A step was added to calculate the fitness, or energy of an ensemble, defined as the sum of total energy over all input states divided by the number of all input states. The ten ensembles with the lowest fitness were used for benchmark analysis.

Single-state design

The protocol for single-state design for each state or PDB file within an ensemble uses the same Rosetta talaris2013 scoring funtion, but each state is designed independently following this protocol:

```
/path/to/rosetta/main/source/bin/rosetta_scripts.linuxgccrelease \\
-s 3c6e_relaxed.pdb -scorefile 3c6e_ssd.fasc

------------------------ssd.options------------------------
-database /path/to/rosetta/main/database
-in:file:fullatom
-in:detect_disulf false
-mute protocols.simple_moves.GenericMonteCarloMover
-parser:protocol ssd.xml
-parser:script_vars resfile=denvE.resfile
-use_input_sc
-linmem_ig 50
-out:file:fullatom
-out:pdb_gz
-out:suffix _ssd_
-nstruct 5
-----------------------------------------------------------
```

```
--------------------------------------ssd.xml--------------------------------------
<ROSETTASCRIPTS>
    <SCOREFXNS>
    </SCOREFXNS>
    <TASKOPERATIONS>
        <InitializeFromCommandline name=ifcl />
        <RestrictToRepacking name=rtr />
        <ReadResfile name=rrf filename=%%resfile%% />
    </TASKOPERATIONS>
    <MOVERS>
        Design movers
        <PackRotamersMover name=design scorefxn=talaris2013 \\
        task_operations=ifcl,rrf />
        <FastRelax name=relax scorefxn=talaris2013 \\
        task_operations=ifcl,rtr repeats=1 />
    </MOVERS>
    <FILTERS>
    </FILTERS>
    <APPLY_TO_POSE>
    </APPLY_TO_POSE>
    <PROTOCOLS>
```

```
            <Add mover=design />
            <Add mover=design />
            <Add mover=design />
            <Add mover=design />
            <Add mover=relax />
      </PROTOCOLS>
</ROSETTASCRIPTS>
```

Generation of sequence profiles using sequences of natural homologues

The following describes the procurement of position-specific scoring matrices (PSSMs), or profiles, of mutation frequencies.

Design profiles

We used the WebLogo tool to generate a fasta file with the sequences of all designs, a sequence logo summarizing the bitscores of all twenty amino acids at each position, and a tab file summarizing the percentage of each amino acid type that populated each designed position. Given the length of the proteins used in the benchmark the sequence logo was not used, whereas the tab file was used for analysis (after first converting the percentages to frequencies).

```
# Generate fasta alignment of each state designed by RECON MSD
cat pdb.list | awk '{system(``design_analysis.py --native ''\$1''.pdb \\
  --format eps --resfile denvE.resfile --multiproc --units probability \\
  designs/''\$1''_msd*pdb'')}'

# Generate fasta alignment of each state designed by SSD
cat pdb.list | awk '{system(``design_analysis.py --native ''\$1''.pdb \\
 --format eps --resfile denvE.resfile --multiproc --units probability \\
 designs/''\$1''_ssd*pdb'')}'
```

```
----------pdb.list----------
1oan_relaxed
1ok8_relaxed
3c5x_relaxed
3c6e_relaxed
3j27_relaxed
3j2p_relaxed
----------------------------
```

PSI-BLAST profiles

PSI-BLAST profiles were generated using a search query of non-redundant sequences using a databased downloaded from the NCBI BLAST server (ftp://ftp.ncbi.nlm.nih.gov/blast/db/) on 2 May, 2014, and and run locally using `psiblast` version 2.2.29 as

```
/path/to/blast/2.2.29/bin/psiblast -query \$NATIVE.fasta -db nr \\
-num_iterations 2 -out \$NATIVE.txt -out_ascii_pssm \$NATIVE.ascii
```

The `$NATIVE.ascii` here represents the generated PSSM of the native sequence derived from the non-redunant database query, which was generated for each PDB native sequence used in the benchmark. The mutation profiles consist of columns 23-42 of the PSSM. Any sequences and the corresponding 20 amino acid type mutation frequencies that did not align to the sequences used for design were manually removed from the generated mutation profile.

Influenza Virus Resource database hemagglutinin stem profiles

Sequences were downloaded on October 14-15, 2019 from the Influenza Virus Resource Database using the search criteria listed in methods for influenza A HA and subtypes H1, H2, H3, H3N2, H4, and H7, as a single file of unaligned FASTA sequences. A multiple sequence alignment was performed using a locally installed Clustal Omega version 1.2.4 as:

```
clustalo -i H3N2.fa -o H3N2_aligned.fa
```

From the multiple sequence alignment, the frequencies of all twenty amino acids present at each aligned position were calculated using WebLogo 3, which was installed locally with Python 3.7,

```
weblogo --sequence-type protein --format logodata --composition none \\
<H3N2_aligned.fa> H3N2.logodata
```

The consensus sequence was determined from the multiple sequence alignment using `EMBOSS v.6.6.0.0` with the `cons` package (ftp://emboss.open-bio.org/pub/EMBOSS/), although was not reported in the manuscript.

```
cons -sequence H3N2_aligned.fa -outseq H3N2.cons
```

The above three command line procedures were applied to each influenza A sequence group. It should be noted the sequences included within the entire influenza A FASTA sequences contained approximately 40 amino acids that were designated with amino acid type 'J', indicating an ambiguous designation between leucine or isoleucine, with only one sequence containing one amino acid type 'J' and each J did not occur at the same aligned position. Each 'J' designation was converted to leucine, since a 'J' designation was not allowed in the multiple sequence alignment using Clustal Omega.

The `.logodata` file generated using WebLogo contained the mutation frequencies of each aligned position for the entire HA protomer. HA1 and unaligned HA2 C-terminus residues were removed from each profile such that only positions that aligned with the native sequence of PDB ID 2HMG, chain F, residues 40-153 were included in each profile for analysis.

Calmodulin natural homologue profiles

The calmodulin mutation profile was determined by using the supplementary dataset Dataset_S01.xlsx provided by Halling, D.B. *et al.* (https://doi.org/10.1073/pnas.1600385113), where the accession number and sequence converted to FASTA format for alignment. The same command line procedures used to obtain HA (sub)type A profiles were used to obtain the calmodulin mutation profile.

Design analysis

Native sequence recovery

The reported native sequence recovery for designs was calculated as the ratio of the native amino acid bit score to all bit scores for each designed position within the ten lowest-scoring models of each designed native PDB model, or $aa_{nat\_frequency} = \frac{bit\_score_{native}}{bit\_score_{all}}$, with

```
python2.7 calc_nat_seq_recovery.py --native 3c6e_relaxed.pdb --nmodels 10 \\
--res denvE.resfile 3c6e_msd_d.tab
```

The above script returns both the native sequence recovery of each designed position as well as the average native sequence recovery. In Fig 3 and in comparisons of sequence recovery to RMSD100, the average native sequence recovery is reported. Otherwise, the native sequence recovery of each designed position, or residue, is reported as a percentage.

The reported native sequence recovery for PSI-BLAST, IVR, and calmodulin profiles was calculated similarly, except that the frequency, not bit score, was used to calculate the native sequence recovery, with $aa_{nat\_frequency} = \frac{frequency_{native}}{frequency_{all}}$. It should be noted that for positions that contained aligned gaps, the $frequency_{all} \neq 1$. The following scripts require a header with the columns labeled for amino acid type, and the first column the native amino acid —labels should be one letter amino acid codes. The first script returns both position-specific native sequence recovery frequencies and average native sequence recovery, and is specific to the `*.ascii` file format. The second script returns only the average native sequence recovery.

```
python calc_pssm_nat_recovery.py -f 3c6e.ascii -o 3c6e_pssm_res_recovery.csv
python calc_msa_nat_recovery.py -f H3N2_align.profile -o H3N2_align_nsr.csv
```

Profile variance

Design mutation preferences, or profiles, were compared to PSI-BLAST profiles by calculating the sum of squared mutation frequency differences between PSI-BLAST and either RECON MSD or SSD profiles, and then normalized by the length of the aligned sequences. The reported average total variance in Fig 3B and Fig 5B represents the average sum of mutation preference differences squared an individual residue exhibits between two profiles, with a score of 0 indicating that the mutation profiles are identical. Unlike when calculating native sequence recovery, comparison of profiles were not calculated for each individual conformation. Instead, the mutation tolerances of each aligned position were averaged using all conformations within the ensemble first before calculating the total variance. Average total variance was calculated using the following

```
python profile_variances.py --reference_profile denvE.ascii \\
--comparison_profile denvE_msd_d.tab --variation_output denvE_RECON_var.csv
```

Testing for equality of variances between two profiles was achieved by using the scipy.stats.levene function, with the center set to median.

```
python Levene_test_for_equal_variances.py profile1.tab profile2.tab
```

Amino acid exchangeability

In the manuscript we used the term *amino acid exchangeability* to represent the averge frequency the native, or i, amino acid is replaced with a non-native, or j, mutation. Average mutation frequencies, including native amino acid conservation frequencies, were calculated by averaging each of the twenty $i \rightarrow j$ mutation frequencies for each i amino acid, using the script below. This script requires a space-delimited file containing a matrix of $n \times m$, with n being the length of all designed positions and m being the mutation profile of the native amino acid, with the first column containing the i amino acid (one-letter code), and a header of the j amino acid frequency columns. Average mutation frequencies were calculated for each conformation to generate a 20×20 matrix of average $i \times j$ frequencies. In the manuscript, we report the average of all $i \times j$ frequencies, which was calculated by taking the cumulative average of all $i \rightarrow j$ frequencies within PSI-BLAST, RECON MSD, and SSD profiles.

```
python per_restype_mutations.py 3c6e_msd_d.tab
```

From the average $i \rightarrow j$ mutation frequencies, all $i \rightarrow j$ frequencies where $j = i$ were excluded to calculate the mean amino acid exchangeability and mean native amino acid exchangeability reported in Fig 4B and Fig 4C. The reported mean amino acid exchangeability represents the average frequency any native amino acid is exchanged for a non-native amino acid; both the mean of amino acid exchangeability rates of all PSI-BLAST, RECON MSD, and SSD profiles and comparison of means were calculated using R.

```{r}
Fig4Bdata <- read.csv("exchangeability_density.csv", header = T, sep = ",")

PSIexchange <- subset(exchange.density, Profile == "PSI-BLAST")
RECONexchange <- subset(exchange.density, Profile == "RECON")
SSDexchange <- subset(exchange.density, Profile == "SSD")

wilcox.test(PSIexchange$Design, RECONexchange$Design,\\
alternative = "two.sided")
wilcox.test(PSIexchange$Design, SSDexchange$Design, \\
alternative = "two.sided")
t.test(PSIexchange$Design, RECONexchange$Design, paired=T, \\
alternative = "two.sided")
t.test(PSIexchange$Design, SSDexchange$Design, paired=T, \\
alternative = "two.sided")

describeBy(exchange.density, group = exchange.density$Profile, mat = T)
```

The mean native amino acid exchangeability rates represents the average frequency a particular native amino acid is exchanged for a non-native amino acid. In Fig 4C, the mean native amino acid exchangeability is reported as the difference between PSI-BLAST mean native amino acid exchangeability rates and either RECON MSD or SSD rates. Individual $i \rightarrow j$ exchangeability frequencies obtained cumulatively from PSI-BLAST, RECON MSD, and SSD are reported in S3 Fig. Kendall τ_{β} and linear regression models were built using R.

```{r}
RECONexchange.corr <- read.csv("RECONexchangeability_correlation.csv",\\
header = T, sep = ",")
RECONexchange.lm <- lm(Design ~ PSIBLAST, data = RECONexchange.corr)
Kendall(RECONexchange.corr$Design, RECONexchange.corr$PSIBLAST)
summary(RECONexchange.lm)

SSDexchange.corr <- read.csv("SSDexchangeability_correlation.csv",\\
header = T, sep = ",")
SSDexchange.lm <- lm(Design ~ PSIBLAST, data = SSDexchange.corr)
Kendall(SSDexchange.corr$Design, SSDexchange.corr$PSIBLAST)$
summary(SSDexchange.lm)
```

Calculation of $RMSD_{da}$ and contact proximity deviation

As in the case of RSV F protein, even though designs contained the same number of residues, not all conformations contained equivalent chain breaks. Therefore, for residues that form either the N- or C-termini of a chain in any conformation were given an $RMSD_{da}$ score of 0. Otherwise, the dihedral angle deviation of a single residue was calculated as described in the **Methods** section.

```
python find_dihedral_deviation.py --list_of_pdb_files 1oan_relaxed.pdb\\
1ok8_relaxed.pdb 3c5x_relaxed.pdb 3c6e_relaxed.pdb 3j27_relaxed.pdb\\
3j2p_relaxed.pdb --output_file denvE_rmsdda.csv
```

Contact proximity deviation was calculated for all aligned positions within an ensemble using the following:

```
python find_contact_deviations.py --pdb_list 1oan_relaxed.pdb \\
1ok8_relaxed.pdb 3c5x_relaxed.pdb 3c6e_relaxed.pdb 3j27_relaxed.pdb \\
3j2p_relaxed.pdb --deviation_matrix denvE_contact_dev.csv \\
--contact_deviation denvE_contact_tally.csv
```

The output within `denvE_rmsdda.csv` and `denvE_contact_tally.csv` were transposed and combined into a single file containing each aligned residue within all eight protein ensembles. A z-score was calculated for each residue's $RMDS_{da}$ and contact proximity score within a single ensemble to normalize scores for all eight ensembles. To calculate the dependency of native sequence recovery on either $RMSD_{da}$ or contact proximity deviation, the average conservation frequency of the native amino acid sequence within each ensemble was used as the reported native residue sequence recovery. Either $RMSD_{da}$ or contact proximity deviationA Kendall's τ_{β} coefficient was calculated using the combined z-scores of all eight ensembles

Plots

The following R scripts were used to generate the figures reported in the manuscript. Note that astericks indicating significance were added using Adobe Illustrator after the initial figure was generated.

Figure 3

```
```{r}
Fig3Adata <- read.csv("benchmark_NSR.csv", header = T, sep = ",")

Fig3A <- ggplot(subset(Fig3Adata, Minimization != "Unminimized"), \\
aes(x = Design, y = Percent_Nat_Seq))
+ geom_boxplot(aes(fill = Design), position=position_dodge(width=0.8))
+ scale_fill_manual(name = "", values=c("black", "#1e90ff", "#ff901e"))
+ labs(x = "", y = "\nNative Sequence Recovery (%)")
+ scale_y_continuous(expand=c(0,0), limits = c(0, 130),breaks=seq(0, 100, by=25))
+ theme(legend.position = "none")

Fig3Bdata <- read.csv("Profile_variability.csv", header = T, sep = ",")

Fig3Bdata$Protein2 <- factor(Fig6Bdata\$Protein, \\
labels = c("5t'-nucleotidase", "Adenylate kinase", "CagL", \\
"Calmodulin","Dengue E protein", "Influenza HA2", "GroEL", \\
"RSV F protein"))

Fig3B <- ggplot(subset(Fig2Bdata, Minimization == "Relaxed"),
aes(x = Design, y = FreqVariability, color=Design, shape=Protein2))
+ geom_point()
+ geom_path(aes(group=Protein2), color="#909090")
+ scale_color_manual(values = c("#1e90ff","#ff901e"), name = "", guide=F)
+ scale_shape_manual(name = "Benchmark Case",
values = c(17, 0, 4, 8, 9, 11, 13, 15, 2, 3))
+ labs(y = "\nNormalized Variability\nfrom PSI-BLAST profile", x="")
+ theme(legend.position = "right", legend.justification = "center",
legend.direction = "horizontal", legend.box = "vertical")
+ guides(shape=guide_legend(ncol = 1 ,byrow=TRUE, title.position = "top"))
+ ylim(0, 1.25)
```
```

```
Fig3 <- plot_grid(Fig3A, Fig3B, nrow = 1, rel_widths = c(0.75, 1),
labels = c("A", "B"))
Fig3
```

Figure 4

Fig 4A PSI-BLAST average mutation frequencies

```{r}
PSIBLAST <- read.csv("n10_AA_freq.csv", header = T, sep = ",")

PSIBLAST$Native <- factor(PSIBLAST$Native, \\
c("G","A","V","L","I","M","F","W","P","S", \\
"T","C","Y","N","Q","D","E","K","R","H"))

PSIBLAST$Mutate <- factor(PSIBLAST$Mutate, \\
c("G","A","V","L","I","M","F","W","P","S", \\
"T","C","Y","N","Q","D","E","K","R","H"))

PSIBLAST <- ggplot(PSIBLAST, aes(Native,Mutate))
+ geom_tile(aes(fill=frequency), color="black")
+ scale_fill_gradientn(name="Mutation\nFrequency", \\
colours=c("white","#ff901e", "#1e90ff","black"), limits = c(0,1), \\
guide = guide_legend(reverse = T), \\
breaks = c(0,0.1,0.2,0.3,0.4,0.5,0.6,0.7,0.8,0.9,1.0))
+ labs(title = "PSI-BLAST")
+ xlab(bquote('Native ' ~ AA[x]))
+ ylab(bquote('Average mutation frequency of ' ~ AA[x]))
+ theme(legend.position = "left", legend.justification = "center")

# Isolate legend for final figure
Fig4Alegend <- get_legend(PSIBLAST)

# Remove legend from panel
PSIBLASTplot <- ggplot(PSIBLAST, aes(Native,Mutate))
+ geom_tile(aes(fill=frequency), color="black")
+ scale_fill_gradientn(name="Frequency", \\
colours=c("white","#ff901e", "#1e90ff","black"), limits = c(0,1), \\
guide = guide_legend(reverse = T), \\
breaks = c(0,0.1,0.2,0.3,0.4,0.5,0.6,0.7,0.8,0.9,1.0))
+ labs(title = "PSI-BLAST")
+ xlab(bquote('Native ' ~ AA[x]))
+ ylab(bquote('Average mutation frequency of ' ~ AA[x]))
+ theme(legend.position = "none")
```

Fig 4A RECON average mutation frequencies

```{r}
RECON <- read.csv("rMSD_AA_freq.csv", header = T, sep = ",")

RECON$Native <- factor(RECON$NativeAA, \\
c("G","A","V","L","I","M","F","W","P","S", \\
```

```
"T","C","Y","N","Q","D","E","K","R","H"))

RECON$Mutate <- factor(RECON$MutateAA, \\
c("G","A","V","L","I","M","F","W","P","S", \\
"T","C","Y","N","Q","D","E","K","R","H"))

RECONplot <- ggplot(RECON, aes(Native,Mutate))
+ geom_tile(aes(fill=frequency), color="black")
+ scale_fill_gradientn(name="", \\
colours=c("white", "#ff901e", "#1e90ff","black"), \\
limits = c(0,1), guide = guide_legend())
+ labs(title = "RECON")
+ xlab(bquote('Native ' ~ AA[x]))
+ ylab(bquote('Average mutation frequency of ' ~ AA[x]))
+ theme(legend.position = "none")
```

Fig 4A SSD average mutation frequencies

```{r}
SSD <- read.csv("rSSD_AA_freq.csv", header = T, sep = ",")

SSD$Native <- factor(SSD$NativeAA, \\
c("G","A","V","L","I","M","F","W","P","S", \\
"T","C","Y","N","Q","D","E","K","R","H"))

SSD$Mutate <- factor(SSD$MutateAA, \\
c("G","A","V","L","I","M","F","W","P","S", \\
"T","C","Y","N","Q","D","E","K","R","H"))

SSDplot <- ggplot(SSD, aes(Native,Mutate))
+ geom_tile(aes(fill=frequency), color="black")
+ scale_fill_gradientn(name="", \\
colours=c("white","#ff901e", "#1e90ff","black"), \\
limits = c(0,1), guide = guide_legend())
+ labs(title = "SSD")
+ xlab(bquote('Native ' ~ AA[x]))
+ ylab(bquote('Average mutation frequency of ' ~ AA[x]))
+ theme(legend.position = "none")
```

Fig 4B

```{r}
Fig4Bdata <- read.csv("exchangeability_density.csv", header = T, sep = ",")

Fig4Bdata$Profile <- factor(exchange.density$Profile,
labels = c("PSI-BLAST", "RECON", "SSD"))

Fig4B <- ggplot(Fig4Bdata, aes(x=Profile,y=Design, fill=Profile))
+ geom_boxplot()
+ scale_fill_manual(name="", values = c( "black","#1e90ff","#ff901e"), guide=F)
+ labs(y="\nExchangeability", x="")
+ theme(axis.text.x = element_text(angle = 45, hjust = 1))
```

```
+ ylim(0, 0.25)
```

Fig 4C

```{r}
Fig4Cdata <- read.csv("AA_freq_avgdeviationCompare_noDesign.csv",
header = T, sep = ",")

Fig4Cdata$Native <- factor(nonNativeDiff$NativeAA, \\
c("G","A","V","L","I","M","F","W","P","S", \\
"T","C","Y","N","Q","D","E","K","R","H"))

Fig4Cdata$profile <- factor(nonNativeDiff$Profile, c("PR", "PS"))

Fig4Cdata$profile <- factor(nonNativeDiff$profile, \\
labels = c("PSI-BLAST - RECON      ", "PSI-BLAST - SSD      "))

Fig4C <- ggplot(Fig4Cdata, aes(x = Native, y = NonNative, \\
color=profile, shape=profile)) + geom_point(size=3)
+ scale_color_manual(values = c("#454545", "#909090"), name = "")
+ scale_shape_manual(name="", values = c(15, 17))
+ xlab(bquote('Native ' ~ AA[average]))
+ ylab(TeX("$\\Delta \\bar{AA}_{exchangeability}$"))
+ ylim(-0.035, 0.035)
+ geom_hline(yintercept = 0, linetype="dashed")
+ theme(legend.position = "bottom", legend.justification = "center", \\
legend.box = "vertical", legend.text = element_text(size = 12), \\
legend.title = element_blank())
+ guides(shape=guide_legend(nrow=1,byrow=TRUE, title.position = "top"))
```

Fig 4

```{r}
Fig4A <- plot_grid(Fig4Alegend, PSIBLAST, RECON, SSD,
nrow = 1, rel_widths = c(0.4,1,1,1), labels = c("A","","",""))

Fig4BC <- plot_grid(Fig4B, Fig4C, nrow = 1, rel_widths = c(0.65, 1),
labels = c("B","C"))
+ theme(plot.margin=unit(c(5.5,5.5,11,5.5), "pt"))

Fig4 <- plot_grid(Fig4A, Fig4BC, ncol = 1)

Fig4
```

Figure 5

Fig 5A and 5C

```{r}
Fig5ACdata <- read.csv("functional_profile_variances.csv", header = T, sep = ",")
```

```
Fig5ACdata$Protein <- factor(funcprofilevar$Protein, \\
labels = c("Calmodulin", "Influenza A HA2"))

calmodulin <- subset(Fig5ACdata, Protein=="Calmodulin")

HA2 <- subset(Fig5AC, Protein=="Influenza A HA2")

calmodulinvar <- ggplot(calmodulin, aes(group=Protein, x=Comparison, \\
y=Deviation, fill=Comparison))
+ geom_bar(stat = "identity", color="black")
+ scale_fill_manual(values = c("#1e90ff","#ff901e"), name = "")
+ labs(y = "\nRMSD from Design Profile", x="")
+ theme(legend.position = "none")
+ ylim(0, 1)
+ facet_grid(~ Protein)

HA2var <- ggplot(HA2, aes(group=Protein, x=Comparison, \\
y=Deviation, fill=Comparison))
+ geom_bar(stat = "identity", color="black")
+ scale_fill_manual(values = c("#1e90ff","#ff901e"), name = "")
+ labs(y = "\nRMSD from Design Profile", x="")
+ theme(legend.position = "none")
+ ylim(0, 1)
+ facet_grid(~ Protein)
```

Fig 5B and 5D

```{r}
calmodulin_resdev <- ggdraw() + draw_image("calmodulin_resdev.png")

HA2_resdev <- ggdraw() + draw_image("influenza_pre_resdev.png")
```

Fig 5

```{r}
plot_grid(calmodulinvar, calmodulin_resdev, HA2var, HA2_resdev, \\
nrow = 2, rel_widths = c(0.4, 1), labels = "AUTO")
```

Figure 6

Fig 6A

```{r}
Fig6Adata <- read.csv("HA_var_dist_matrix.csv", header = T, sep = ",")

Fig6Adata.matrix <- as.matrix(Fig6Adata[,-c(1)])

rownames(Fig6Adata.matrix) <- Fig6Adata$Subtype

Fig6A.dendro <- as.dendrogram(hclust(d = dist(x = Fig6Adata.matrix)))
```

```
HAdendsort <- dendsort(Fig6A.dendro)

plot(HAdendsort, type = "triangle")
```

Fig 6B

```{r}
Fig6Bdata <- read.csv("HA2_subtype_variances.csv", header = T, sep = ",")

Fig6Bdata$Subtype <- factor(HA2subtypesdata$Subtype, \\
c("H3","H3N2","H4","H7","H1","H2"))

Fig6B <- ggplot(Fig6Bdata, aes(x=Subtype, y=RMSD, fill=Comparison))
+ geom_bar(stat = "identity", color="black", position=position_dodge())
+ scale_fill_manual(values = c("#1e90ff","#ff901e"), \\
name = "IVR MSA profile RMSD with respect to")
+ labs(y = "\nRMSD", x="IVR MSA Profile") + ylim(0, 1)
+ theme(legend.position = "bottom")
```

Figure 7

Fig 7A

```{r}
Fig7Adata <- read.csv("benchmark_relaxed_NSR.csv", header = T, sep = ",")

Fig7Adata$maxRMSD100 <- cut2(Fig7Adata$MaxRMSD100, g=3)

Fig7A <- ggplot(relaxnsr, aes(x = maxRMSD100, y = Percent_Nat_Seq))
+ geom_boxplot(aes(color=Design, fill=Design), alpha=0.3)
+ labs(x = "\nMaximum RMSD100 (Å)", y = "\nNSR (\%)")
+ scale_color_manual(name = "", values = c("black","dodgerblue","#ff901e"))
+ scale_fill_manual(name = "", values = c("black","dodgerblue","#ff901e"))
+ facet_wrap(~ Design)
+ theme(legend.position = "none", \\
axis.text.x = element_text(size = 8, angle = 45, hjust = 1))
+ ylim(0,130)
```

Fig 7B

```{r}
Fig7BCdata <- read.csv("proximity_diRMSD_bydesign.csv", header = T, sep = ",")

Fig7BC$Measure <- factor(Fig7BCdata$Measure, labels = c("Contact Proximity\n \\
Deviation (Å)", "Dihedral Angle\nDeviation (rad)"))

Fig7Bdata <- subset(Fig7BCdata,Measure == "Contact Proximity\nDeviation (Å)")

Fig7Bdata$deviation <- cut2(Fig7Bdata$Deviation, g=3)

Fig7B <- ggplot(Fig7Bdata, aes(x=deviation, y=AvgPNSR, color=Design))
```

```
+ geom_boxplot(aes(color=Design, fill=Design), alpha=0.3)
+ scale_color_manual(name="", values = c("black","#1e90ff","#ff901e"))
+ scale_fill_manual(name="", values = c("black","#1e90ff","#ff901e"))
+ labs(x="\nContact Proximity Deviation z-score (Å)", y="\nResidue NSR (\%)")
+ theme(axis.text.x = element_text(angle = 45, size = 10, hjust = 1))
+ ylim(0,130)
+ facet_wrap(~ Design)
+ theme(legend.position = "none", \\
axis.text.x = element_text(size = 8, angle = 45, hjust = 1))
```

Fig 7C

```{r}
Fig7Cdata <- subset(Fig7BCdata,Measure == "Dihedral Angle\nDeviation (rad)")

Fig7C$deviation <- cut2(Fig7Cdata$Deviation, g=3)

Fig7Cdata <- ggplot(Fig7Cdata, aes(x=deviation, y=AvgPNSR, color=Design))
+ geom_boxplot(aes(color=Design, fill=Design), alpha=0.3)
+ scale_color_manual(name="", values = c("black","#1e90ff","#ff901e"))
+ scale_fill_manual(name="", values = c("black","#1e90ff","#ff901e"))
+ labs(x="\nDihedral Angle RMSD z-score (rad)", y="\nResidue NSR (\%)")
+ theme(axis.text.x = element_text(angle = 45, size = 10, hjust = 1))
+ facet_wrap(~ Design)
+ theme(legend.position = "none", axis.text.x = element_text(size = 8, \\
angle = 45, hjust = 1))
+ ylim(0,130)
```

Figure 8

```{r}
Fig8data <- read.csv("benchmark_energies_relaxed-w-templates.csv",
header = T, sep = ",")

Fig8data$Design <- factor(Fig8data$Design, labels = c("RECON","SSD","Native"))

Fig8data$Dataset2 <- factor(Fig8data$Dataset, \\
labels = c("5t'-nucleotidase", "Adenylate kinase", "CagL", \\
"Calmodulin","Dengue E protein","Influenza HA stem", \\
"GroEL subunit", "RSV F protein"))

Fig8 <- ggplot(Fig7data, aes(x = Design, y = ResScore))
+ geom_violin(aes(fill = Design))
+ labs(x = "", y = "Mean Residue Score (REU)")
+ scale_fill_manual(name="", values = c("#1e90ff","#ff901e","grey50"))
+ facet_wrap(~ Dataset2, ncol = 4)
+ theme(axis.text.x = element_blank(), axis.ticks.x = element_blank())
+ ylim(-2.5, -1.0)
```

Figure 9

Fig 9A

```
```{r}
Fig9Adata <- read.csv("benchmark_energies_relaxed-w-templates.csv",\\
header = T, sep = ",")

Fig9Adata$maxRMSD100 <- cut2(Fig9Adata$MaxRMSD100, g=3)

Fig9Adata$Design <- factor(Fig9Adata$Design, labels = c("RECON","SSD","Native"))

Fig9Adata$Design <- factor(Fig9Adata$Design, c("Native","RECON","SSD"))

Fig9Adata.subset <- subset(Fig9Adata, Design != "Native")

Fig9A <- ggplot(Fig9Adata.subset, aes(x=maxRMSD100, y=ResScore))
+ geom_boxplot(aes(color=Design, fill=Design), alpha=0.3)
+ labs(x = "\nMaximum RMSD100 (Å)", y = "\nMean Residue Total Score (REU)")
+ scale_color_manual(name = "", values = c("#1e90ff","#ff901e"))
+ scale_fill_manual(name = "", values = c("#1e90ff","#ff901e"))
+ facet_wrap(~ Design)
+ theme(legend.position = "none", \\
axis.text.x = element_text(size = 6, angle = 45, hjust = 1), \\
axis.title = element_text(size = 10), title = element_text(size = 12))
+ ylim(-5,2.5)
```
```

Fig 9B

```
```{r}
Fig9Bdata <- read.csv("proximity_diRMSD_bydesignonly.csv", header = T, sep = ",")

Fig9Bdata$Measure <- factor(Fig9Bdata$Measure, \\
labels = c("Contact Proximity\nDeviation (Å)", "Dihedral Angle\nDeviation (rad)"))

Fig9Bdata <- subset(Fig8Bdata, Measure == "Dihedral Angle\nDeviation (rad)")

Fig9Bdata$deviation <- cut2(Fig9Bdata$Deviation, g=3)

Fig9B <- ggplot(Fig9Bdata, aes(x=deviation, y=ResEnergy, color=Design))
+ geom_boxplot(aes(color=Design, fill=Design), alpha=0.3)
+ scale_color_manual(name="", values = c("#1e90ff", "#ff901e"))
+ scale_fill_manual(name="", values = c("#1e90ff", "#ff901e"))
+ labs(x="\nDihedral Angle RMSD z-score (rad)", y="Residue Score (REU)")
+ facet_wrap(~ Design)
+ theme(legend.position = "none", \\
axis.text.x = element_text(size = 6, angle = 45, hjust = 1), \\
axis.title = element_text(size = 10), title = element_text(size = 12))
+ ylim(-5, 2.5)
```
```

Fig 9C

```
```{r}
```
```

```
Fig9Cdata <- read.csv("proximity_diRMSD_bydesignonly.csv", header = T, sep = ",")

Fig9Cdata$Measure <- factor(Fig9Cdata$Measure, \\
labels = c("Contact Proximity\nDeviation (Å)", "Dihedral Angle\nDeviation (rad)"))

Fig9Cdata <- subset(Fig8Cdata,Measure == "Contact Proximity\nDeviation (Å)")

Fig9Cdata$deviation <- cut2(Fig9Cdata$Deviation, g=3)

Fig9C <- ggplot(Fig9Cdata,  aes(x=deviation, y=ResEnergy, color=Design))
+ geom_boxplot(aes(color=Design, fill=Design), alpha=0.3)
+ scale_color_manual(name="", values = c("#1e90ff", "#ff901e"))
+ scale_fill_manual(name="", values = c("#1e90ff", "#ff901e"))
+ labs(x="\nContact Proximity Deviation (Å)", y="Residue Score (REU)")
+ facet_wrap(~ Design)
+ theme(legend.position = "none", \\
axis.text.x = element_text(size = 6, angle = 45, hjust = 1), \\
axis.title = element_text(size = 10), title = element_text(size = 12))
+ ylim(-5, 2.5)
```
```

## S1 Fig

S1 Fig A

```{r}
S1Adata <- read.csv("benchmark_NSR.csv", header = T, sep = ",")

S1AFig <- ggplot(S1Adata, aes(x = Minimization, y = Percent_Nat_Seq))
+ geom_boxplot(aes(fill = Design), position = position_dodge(width=0.8))
+ facet_grid(cols = vars(Design), scales = "free_x", space = "free_x")
+ scale_fill_manual(name = "", values = c("black","#1e90ff", "#ff901e"))
+ labs(x = "", y = "\nNative Sequence Recovery (%)")
+ scale_y_continuous(expand=c(0,0), limits = c(0,130), breaks=seq(0,100,by=25))
+ theme(legend.position = "none")
```

S1 Fig B

```{r}
S1Bdata <- read.csv("Profile_variability.csv", header = T, sep = ",")

S1Bdata$Protein2 <- factor(S1Bdata$Protein, labels = c("5t'-nucleotidase",\\
"Adenylate kinase", "CagL", "Calmodulin","Dengue E protein", "Influenza HA2",\\
"GroEL", "RSV F protein"))

S1BFig <- ggplot(S1Bdata, aes(x = Design, y = FreqVariability, color=Design,\\
shape=Protein2))
+ geom_point()
+ geom_path(aes(group=Protein2), color="#909090")
+ scale_color_manual(values = c("#1e90ff", "#ff901e"), name = "", guide=F)
+ scale_shape_manual(name = "Benchmark Case", values = c(17,0,4,8,9,11,13,15,2,3))
+ facet_grid(~ Minimization)
```
```

```
+ labs(y = "\nNormalized Variability\nfrom PSI-BLAST profile")
+ theme(axis.title.x = element_blank(), legend.position = "bottom", \\
legend.justification = "center", legend.direction = "horizontal", \\
legend.box = "vertical")
+ guides(shape=guide_legend(nrow=4,byrow=TRUE, title.position = "top"))
+ ylim(0, 1.25)
```

S1 Fig

```{r}
S1Fig <- plot_grid(S1AFig, S1BFig, nrow = 1, rel_widths = c(1,0.7),\\
labels = c("A","B"))
```

S2 Fig

```{r}
S2data <- read.csv("AA_freq_deviationCompare.csv", header = T, sep = ",")

S2data$Native <- factor(S2data$NativeAA, \\
c("G","A","V","L","I","M","F","W","P","S", \\
"T","C","Y","N","Q","D","E","K","R","H"))

S2data$Mutate <- factor(S2data$MutateAA, \\
c("G","A","V","L","I","M","F","W","P","S", \\
"T","C","Y","N","Q","D","E","K","R","H"))

S2data$profile <- factor(S2data$Profile, c("PR","PS","Design"))

S2data$profile <- factor(S2data$profile,\\
labels = c("PSI-BLAST - RECON", "PSI-BLAST - SSD", "RECON - SSD"))

S2Fig <- ggplot(S2data, aes(Native,Mutate))
+ geom_tile(aes(fill=FrequencyDiff), color="black")
+ scale_fill_gradientn(name=TeX("$\\Delta$ Freq"), \\
colours=c("#994d00","white","#004d99","black"), \\
limits = c(-0.3, 0.6), guide = guide_legend(reverse = T), \\
breaks = c(-0.3,-0.2,-0.1,0.0,0.1,0.2,0.3,0.4,0.5,0.6))
+ facet_grid(~ profile)
+ xlab(bquote('Native ' ~ AA[x]))
+ ylab(TeX("$\\Delta$ Average mutation frequency of $AA_x$"))
+ theme(legend.position = "left", legend.justification = "center" )
```

S3 Fig

S3 Fig A

```{r}
RECONexchange.corr <- read.csv("RECONexchangeability_correlation.csv",\\
header = T, sep = ",")
```

```
RECONexchange.lm <- lm(Design ~ PSIBLAST, data = RECONexchange.corr)

RECONexchange.model <- augment(RECONexchange.lm)

RECONexchange.model$predicted <- predict(RECONexchange.lm)

RECONexchange.model$residuals <- residuals(RECONexchange.lm)

RECONexchange.residuals <- ggplot(RECONexchange.model,aes(x=PSIBLAST,y=Design))
+ geom_point(aes(color = abs(residuals), alpha = abs(residuals)))
+ xlim(0, 0.175)
+ ylim(0, 0.175)
+ geom_abline(intercept = 0, slope = 1, color="#909090")
+ labs(x="PSI-BLAST", y="\nRECON")
+ scale_color_continuous(low = "#1e90ff", high = "black", limit=c(0,0.1))
+ guides(alpha=F, color=F)

SSDexchange.corr <- read.csv("SSDexchangeability_correlation.csv", \\
header = T, sep = ",")

SSDexchange.lm <- lm(Design ~ PSIBLAST, data = SSDexchange.corr)

SSDexchange.model <- augment(SSDexchange.lm)

SSDexchange.model$predicted <- predict(SSDexchange.lm)

SSDexchange.model$residuals <- residuals(SSDexchange.lm)

SSDexchange.residuals <- ggplot(SSDexchange.model,aes(x=PSIBLAST,y=Design))
+ geom_point(aes(color = abs(residuals), alpha = abs(residuals)))
+ xlim(0, 0.175)
+ ylim(0, 0.175)
+ geom_abline(intercept = 0, slope = 1, color="#909090")
+ labs(x="PSI-BLAST", y="\nSSD")
+ scale_color_continuous(low = "#ff901e", high = "black", limit=c(0,0.1))
+ guides(alpha=F, color=F)

```

S3 Fig B

```{r}
RECONexchange.lm <- lm(Design ~ PSIBLAST, data = RECONexchange.corr)

indexlabel <- read.csv("IndexLabel.csv", header = T)

N <- nrow(RECONexchange.lm$model)

df <- data.frame(Index = 1:N,  dfstats = dfbetas(RECONexchange.lm))

df$group <- factor(ifelse(df$dfstats.PSIBLAST > 0.1026 , 1, \\
ifelse(df$dfstats.PSIBLAST < -0.1026 , 1,0)))
```

```
df <- cbind(df, indexlabel)

RECON.dfbeta <- ggplot(df, aes(Index, dfstats.PSIBLAST))
+ geom_point(size=0.25, aes(color=group))
+ geom_segment(aes(Index,xend=Index,0,yend=dfstats.PSIBLAST,color=group),data=df)
+ scale_color_manual(values=c("#1e90ff", "black"))
+ labs(y="\nDFBETA")
+ guides(color = F)
+ ylim(-1,1)
+ geom_label_repel(data = subset(df, group=="1"), aes(label=Labelindex), \\
nudge_y = 0.1, direction="y",segment.color = "grey80", \\
segment.size = 0.5, size = 1.5)

SSDexchange.lm <- lm(Design ~ PSIBLAST, data = SSDexchange.corr)

N <- nrow(SSDexchange.lm$model)

df <- data.frame(Index = 1:N,  dfstats = dfbetas(SSDexchange.lm))

df$group <- factor(ifelse(df$dfstats.PSIBLAST > 0.1026 , 1, \\
ifelse(df$dfstats.PSIBLAST < -0.1026 , 1,0)))

df <- cbind(df, indexlabel)

SSD.dfbeta <- ggplot(df, aes(Index, dfstats.PSIBLAST))
+ geom_point(size=0.25, aes(color=group))
+ geom_segment(aes(Index,xend=Index, 0,yend=dfstats.PSIBLAST,color=group),data=df)
+ scale_color_manual(values=c("#ff901e", "black"))
+ labs(y="\nDFBETA")
+ guides(color = F)
+ ylim(-1,1)
+ geom_label_repel(data = subset(df, group=="1"), aes(label=Labelindex), \\
nudge_y = 0.1, direction="y", segment.color = "grey80", \\
segment.size = 0.5, size = 1.5)
```

S3 Fig

```{r}
S3Fig <- plot_grid(RECONexchange.residuals, SSDexchange.residuals, \\
RECON.dfbeta, SSD.dfbeta, nrow = 2, ncol = 2, labels = c("A","","B",""))
```

S4 Fig

```{r}
S4data <- read.csv("HA_var.csv", header = T, sep = ",")

S4data$Subtype <- factor(S4data$Subtype, c("H3N2","H3","H4","H7","H1","H2"))

ggplot(S4data, aes(x=ResNum, y=ResDev, color=Subtype))
+ geom_point()
```

```
+ scale_color_manual(name="Subtype", values = c("#901eff","#982fea","#a548cc",\\
"#ba7298","#ca9370", "#e3c532"))
+ labs(x="Residiue Number (2HMG, chain F)", \\
y="RMSD of design profile to IVR subtype profile")
+ facet_grid(Subtype ~ Design)
+ theme(legend.position = "bottom", legend.justification = "center", \\
legend.direction = "horizontal", legend.box = "vertical")
+ theme(legend.position = "none")
```

S5 Fig

S5 Fig A

```{r}
S5data <- read.csv("proximityXcontact.csv", header = T, sep=",")

S5AFig <- ggplot(S5data, aes(x=diRMSD, y=Tally))
+ geom_point(color="white", size=0.25) + geom_hex(bins=25)
+ scale_fill_gradientn(name="Residue Count", \\
colours=c("#E8E8E8","#a8a8a8","#909090","#454545","#222222","black"), \\
guide = guide_legend())
+ labs(x="Dihedral Angle\nDeviation (rad)", y="\nContact Proximity\nDeviation (Å)")
+ theme(legend.position = "bottom", \\
legend.justification = "center", legend.direction = "horizontal", \\
legend.box = "vertical", legend.text = element_text(size = 8), \\
legend.key.height = grid::unit(0.33,"cm"), legend.key.width = grid::unit(0.33,"cm"))
+ guides(fill=guide_legend(nrow = 1, byrow = T, title.position = "top", \\
title.hjust = 0.5), color=F)
```

S5 Fig B

```{r}
S5data <- read.csv("proximityXcontact.csv", header = T, sep=",")

S5BFig <- ggplot(S5data, aes(x=ZdiRMSD, y=ZTally))
+ geom_point(color="white", size=0.25) + geom_hex(bins=25)
+ scale_fill_gradientn(name="Residue Count", \\
colours=c("#E8E8E8","#a8a8a8","#909090","#454545","#222222","black"), \\
guide = guide_legend(), breaks = c(10,25,50,75,100,150))
+ labs(x="Dihedral Angle\nDeviation z-score (rad)", \\
y="\nContact Proximity\nDeviation z-score (Å)")
+ theme(legend.position = "bottom", legend.justification = "center", \\
legend.direction = "horizontal", legend.box = "vertical", \\
legend.text = element_text(size = 8),legend.key.height = grid::unit(0.33,"cm"), \\
legend.key.width = grid::unit(0.33,"cm"))
+ guides(fill=guide_legend(nrow = 1, byrow = T, title.position = "top", \\
title.hjust = 0.5), color=F)
```
