# Supplementary figures and images for: Multi-state design of flexible proteins predicts sequences optimal for conformational change
Source: PLoS Comput Biol. 2020 Feb 7;16(2):e1007339. doi: 10.1371/journal.pcbi.1007339 (PMC7032724; doi:10.1371/journal.pcbi.1007339)

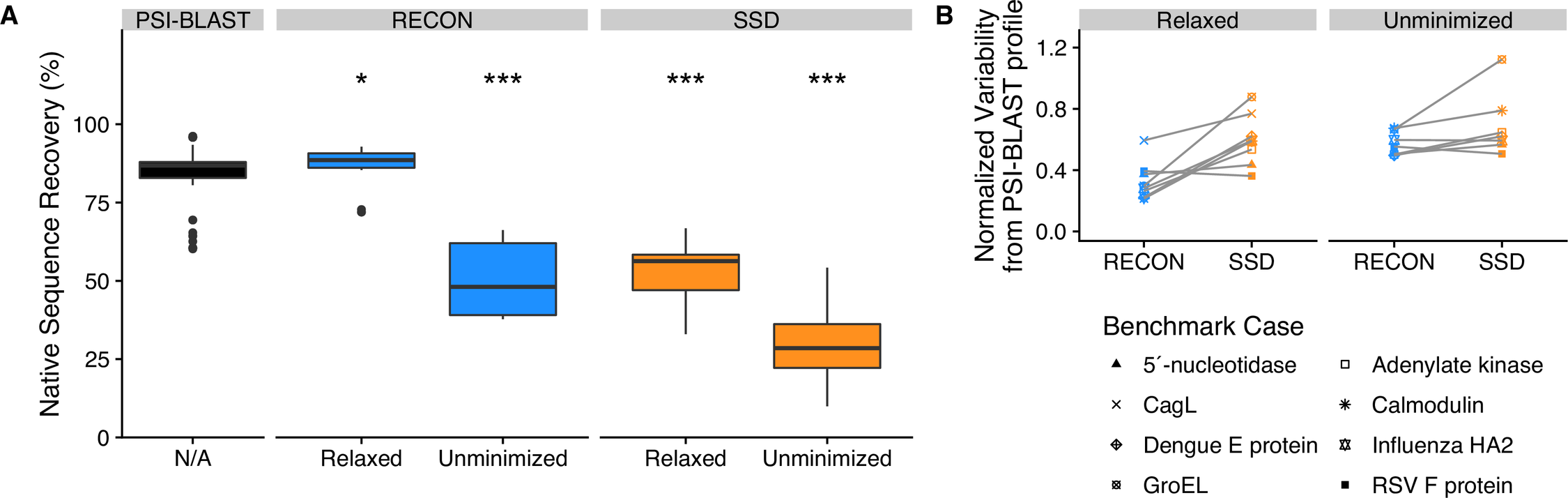

Supplement: S1 Fig — (A) Comparison of total native sequence recovery of relaxed and unminimized RECON MSD and SSD designs to PSI-BLAST sequence profiles generated using the native sequence. Asterisks indicate the significance of difference of means of each design in comparison to the PSI-BLAST profile, with a z-test p-value < 0.01 represented by one asterisk, and a p-value < 0.00001 by three asterisks. (B) Mutation frequency variances of designs in comparison to a PSI-BLAST profile, normalized by protein length. The y-axis values represent the average variability of mutation profiles for each designed residue in relation to a PSI-BLAST profile, as described in Fig 3. (TIF) [file pcbi.1007339.s003.tif]

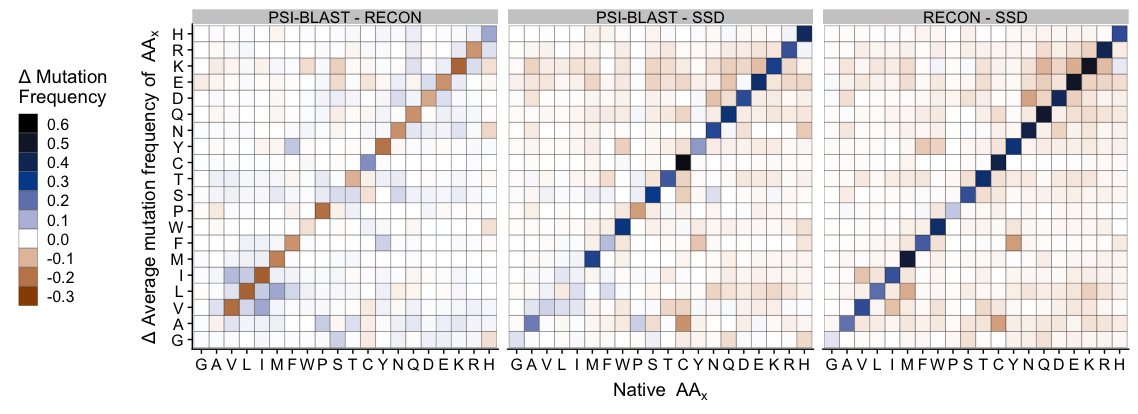

Supplement: S2 Fig — The x axis represents the original amino acid. The y axis represents the difference in average mutation frequencies between two profiles, which are noted above each grid. Along the diagonal axis, indicating native sequence conservation, values less than zero (oranges) signify that the latter profile was more highly conserved, and values greater than zero (blues to black) signify that the native residue was less conserved in the latter profile. Not along the diagonal, values less than zero indicate that exchangeability of the native residue to the indicated residue along the y axis was higher in the latter profile, whereas values greater than zero indicate that exchangeability was lower in the latter profile. (TIF) [file pcbi.1007339.s004.tif]

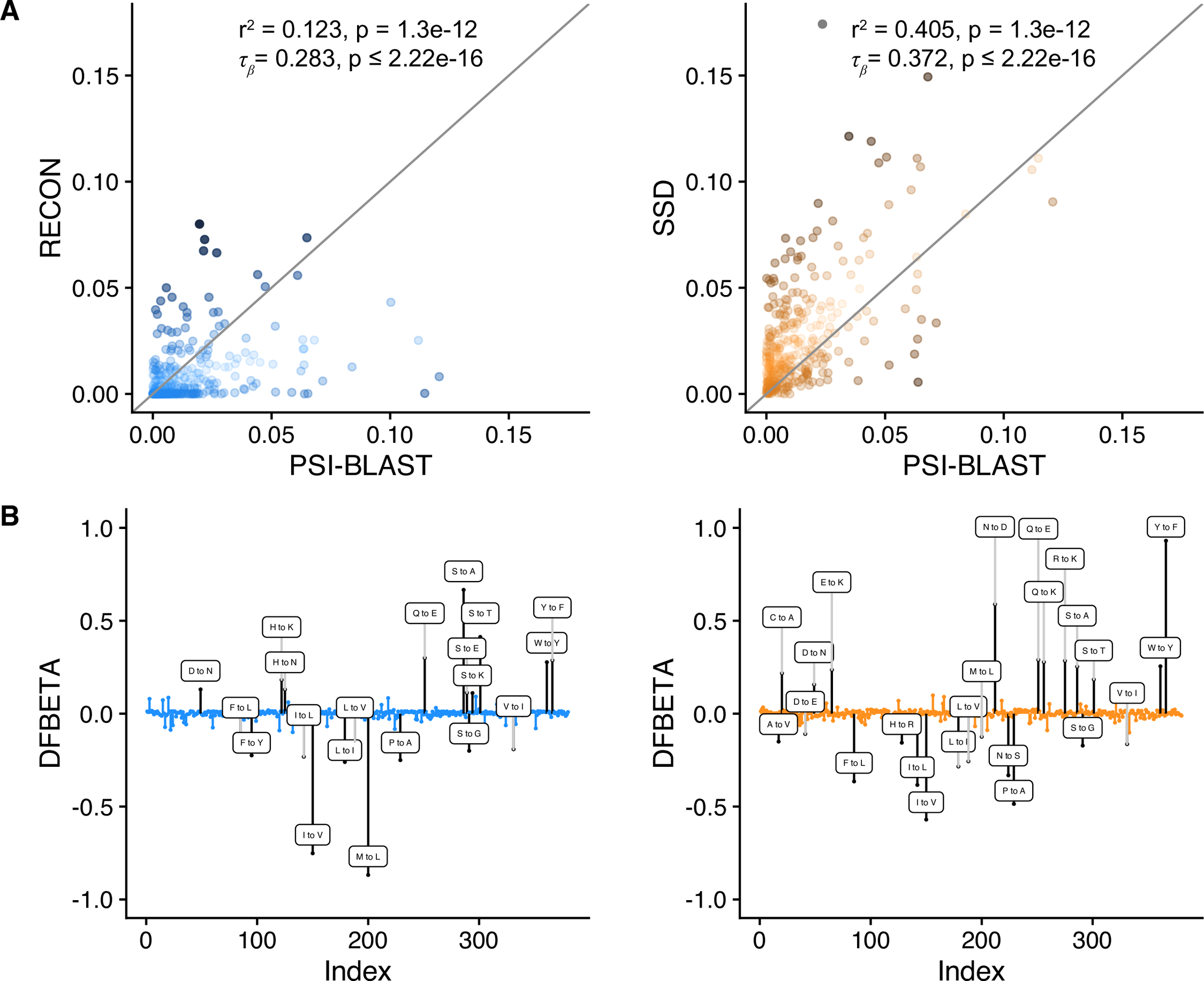

Supplement: S3 Fig — (A) Scatterplots of each exchangeability rate as observed in a PSI-BLAST profile compared to design profile. Both the x and y axes represent the exchangeability frequency of a native amino acid to a specific, non-native amino acid, with PSI-BLAST exchangeability rates along the x axis, and design exchangeability rates along the y axis. For reference, a grey line drawn is drawn along where the exchangeability rates would be equal between a PSI-BLAST and design profile. Both an adjusted r2 and τβ value is provided, along with the associated two-sided p-value. Lighter points are found along the linear regression model, and darker points represent outliers. (B) Measures of influence for individual exchangeability rate. The index listed along the x axis refers to each exchangeability rate, indexed in order alphabetically. For reference, in the first nineteen indices, the first index refers to the A to C mutation frequency, followed by the next eighteen indices that correspond with A to D through Y mutation frequencies. The measure of influential observation, or DFBETA index, is represented along the y axis. The height and direction of each bar corresponds with the change in regression model correlation coefficient without that particular observation. Influential outliers that have >|2N| index value, or ±0.106 threshold, are colored in black and are labeled with the associated mutation. (TIF) [file pcbi.1007339.s005.tif]

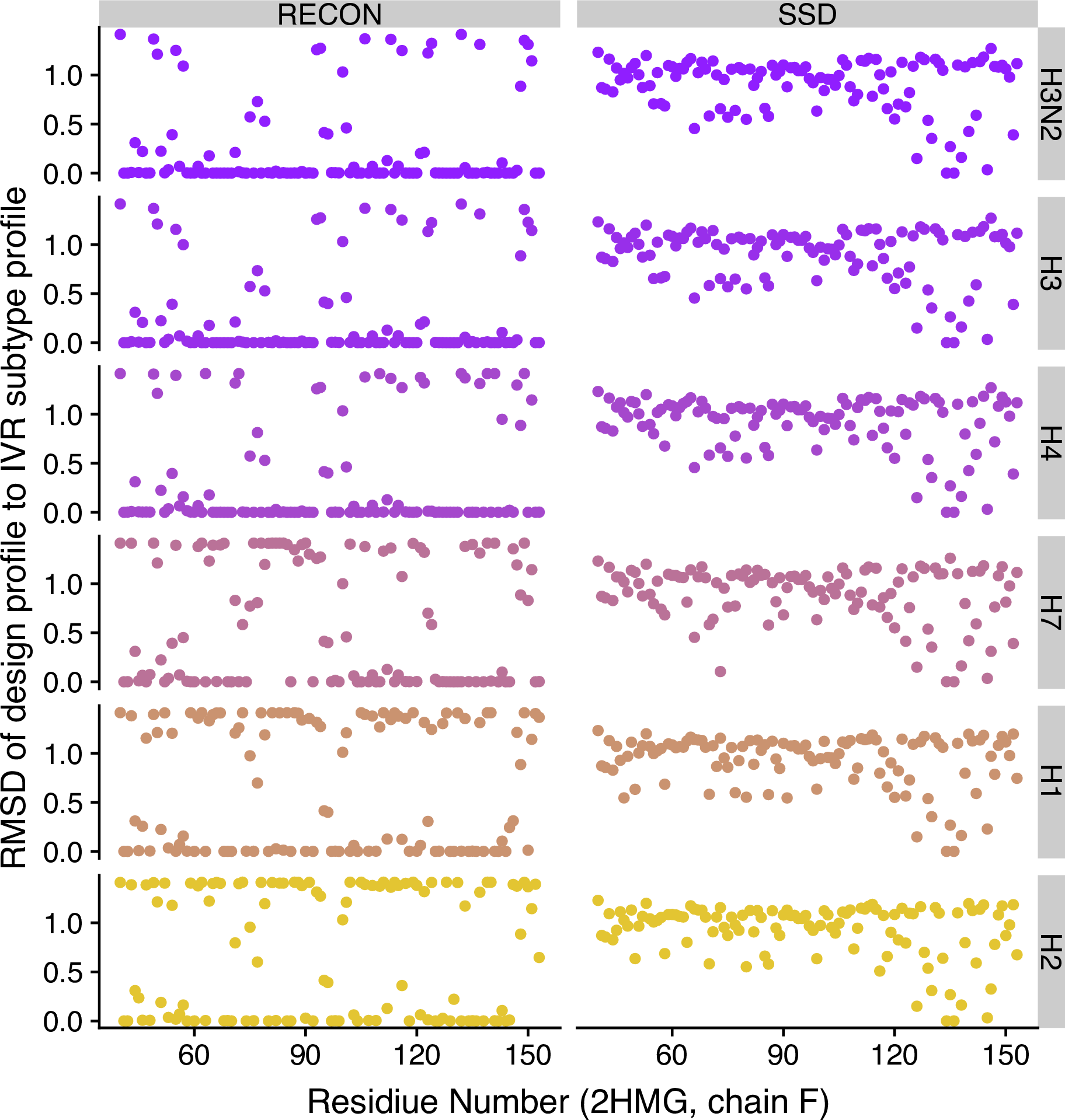

Supplement: S4 Fig — Each IVR subtype mutation profile was generated by multiple sequence alignment of HA2 sequences within the IVR database, subdivided by HA subtype including H1, H2, H3, H4, and H7. Because the designed sequence used only an H3N2 HA2 backbone, the H3N2 subtype was included in addition to H3. Only positions that align to the native sequence used for design were included within the profile. HA2 subsequences are separated and ordered by similarity to H3N2, from highest similarity on the top. The x axis each aligned position of the HA2 sequence, corresponding to the H3N2 residue numbering of PDB ID 2HMG, chain F. The y axis is the root mean square deviation (RMSD) of each residue’s subtype-specific profile within the multiple sequence alignment with respect to RECON MSD, on the left, and to SSD on the right. (TIF) [file pcbi.1007339.s006.tif]

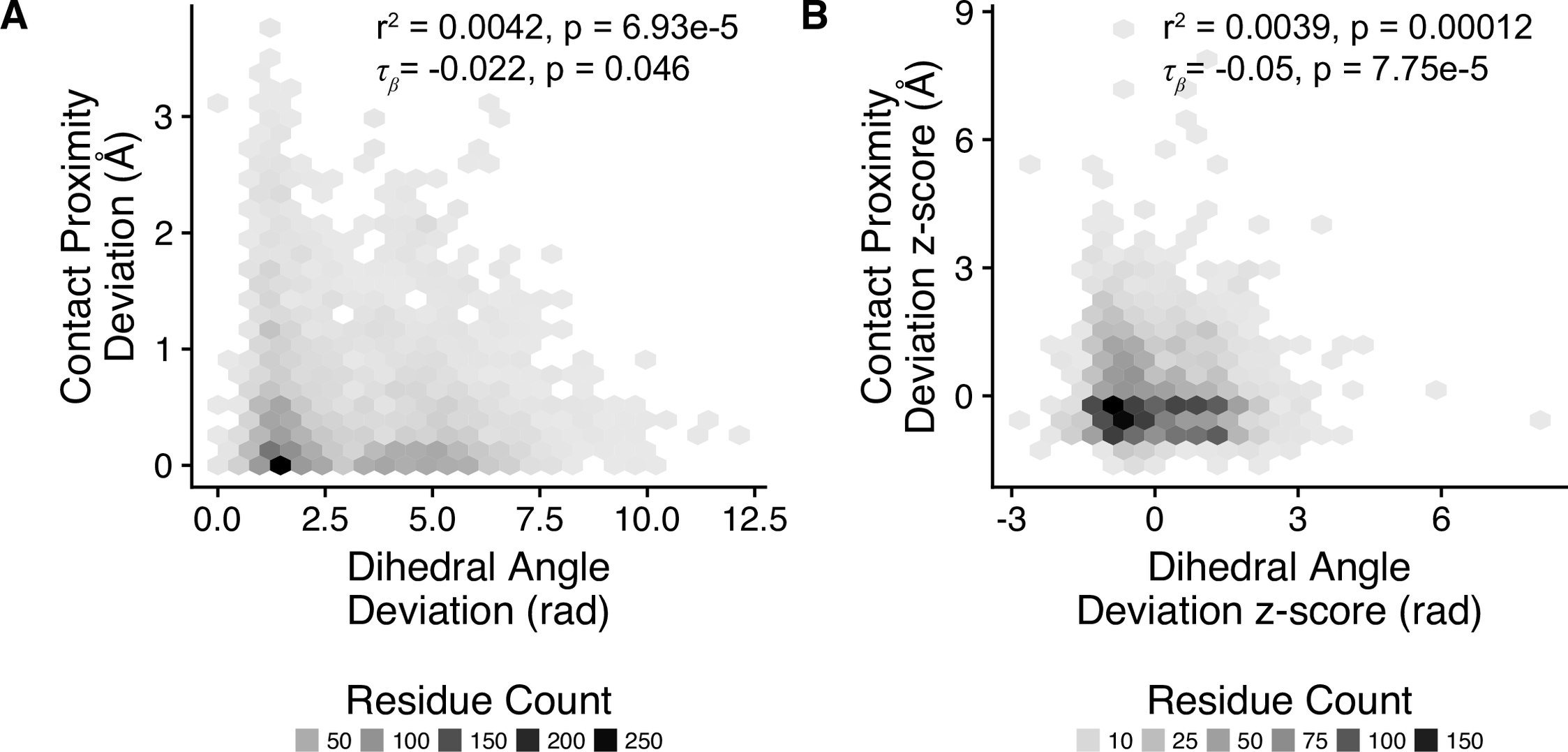

Supplement: S5 Fig — (A) The x-axis represents dihedral RMSD, measured in radians, and the y-axis represents contact proximity deviation, measured in Å. The hex bins shaded in grey are the number of residues within the deposited PDB structure have have both a Cβ–Cβ distance deviation and dihedral angle RMSD within a bin. (B) Axes represent same metrices as in Panel A, normalized by z-score. (TIF) [file pcbi.1007339.s007.tif]
