# Supplementary material for: Multi-state design of flexible proteins predicts sequences optimal for conformational change
Source: PLoS Comput Biol. 2020 Feb 7;16(2):e1007339. doi: 10.1371/journal.pcbi.1007339 (PMC7032724; doi:10.1371/journal.pcbi.1007339)
Supplement: Attachment — Submitted filename: Response_to_Reviewers.docx [file pcbi.1007339.s008.docx]

Dear Professors Wallner and Elofsson,

We have carefully analyzed the reviewer comments and revised our manuscript, entitled “Multi-State Design of Flexible Proteins Predicts Sequences Optimal for Conformational Change”, following their suggestions. We feel that the manuscript has substantially improved. We hope that you will find this revised version acceptable for publication in *PLoS Computational Biology*. Please find a detailed summary of all changes applied to the manuscript and our point-by-point responses to the reviewer comments below.

Best regards,

Marion F. Sauer

We thank the reviewers for their constructive critique. To facilitate re-review of this article, we

include all changes applied to address the reviewer comments as part of this point-by-point

response.

**Reviewer #1:** **The Meiler lab previously described their method for multistate design called RECON and have used it recently in improving the cross-reactivity of an anti-influenza antibody. Very briefly, RECON is more efficient than conventional multistate design algorithms because it uses a heuristic that first samples sequence space across all possible protein states and then defines sequence constraints based on this sampling to encourage the convergence of all states to a single sequence acceptable to all. Here, Sauer et al. benchmark the method's ability to produce sequences of conformationally flexible proteins that are similar to the sequence space of their natural homologs. This problem had been tackled previously as the authors note, for instance by Kortemme's lab, and yet, it is clear that the method used here is, at least in principle, superior to those used previously. Briefly, past applications of multistate design to this problem looked for sequence profiles that are "on average" acceptable across all states independently, whereas here, the sequences are actually tested on each and every state. The authors also describe a new metric for estimating conformational differences between protein states. Although this metric may be novel, they do not present a compelling case for its usefulness.**

*We thank this reviewer for the positive assessment of our manuscript. We rephrased a paragraph in* ***the*** ***Discussion*** *section to better make the case for scenarios where the new metric is useful:*

## Contact proximity deviation captures local and global conformational rearrangements as a single metric

Methods like Local-Global Alignment and contact area differences are useful in circumventing the over-estimation of global structural dissimilarity by either emphasizing local backbone segment structure similarity or by side chain placement similarity, respectively (1, 2). In particular, contact area differences between two homologues have been shown to be as accurate as RMSD, if not more, in comparing the structural similarity of proteins with very high sequence similarity (3). However, contact area differences are sensitive to the placement of side chains within a structural model, so that it becomes difficult to compare conformations that differ in sequence.

To overcome this limitation, we introduced the contact proximity deviation metric (see Methods section). Contact proximity quantifies the relative placement of a residue within a structure and is sensitive to a change in conformation without relying on side-chain contacts. To decide whether two residues are in contact, we analyze the C_β_ − C_β_ distance. However, instead of a hard cutoff distance we use a smooth transition function to avoid discontinuities when distances change by small margins. Contact proximity deviation for a residue becomes the sum of changes in contact proximity when comparing two structures. Therefore, contact proximity deviation quantifies the magnitude of local rearrangements around a residue of interest inflicted by a global conformational change, independent of side chain identity.

Thus, combining the contact proximity deviation metric with interaction network analysis (4) could provide a useful tool to investigate how conformational rearrangements alter residue networks. Additionally, in cases such as the design of protein switches or structural analysis of mutations where protein flexibility, but not sequence, needs to be conserved, it is helpful to have a metric that highlights residues that experience rearrangements in their contacts. Lastly, the measurement of local structural variability by NMR residual dipolar coupling has shown that regions of high flexibility within ubiquitin ensembles align with multiple protein-protein interfaces (5). Thus, contact proximity deviation provides a possible approach to study protein-protein interaction interfaces.

**Major comment:**
**1. Throughout the paper, the authors point out that RECON is more conservative in mutations than alternative single-state design methods. This makes a lot of sense and is expected. The problem is that the benchmark provided in the paper -- the designs' compatibility with the natural homologs' sequence diversity -- may therefore be misleading. The most conservative design method, i.e., consensus design, would probably be even superior to RECON according to this metric. The main advantage of a design algorithm is obviously if it can come up with sequence choices that are non-trivial, that is, they don't appear in the natural diversity and yet are compatible with function. Given that there are now large deep mutational scanning datasets for many proteins, it seems natural to test whether RECON "predicts" these data. I hardly expect a perfect prediction (this is a very tough challenge), but an improvement relative to PSI-BLAST and single-state design methods is expected. Of course, successful prediction in this case would also make the claim of usefulness much stronger as these deep sequencing analyses are expensive and time-consuming***.*

*This is an excellent suggestion. We substantially revised the manuscript and added this analysis. The following was added to the* ***Results*** *section:*

## RECON MSD prediction of mutation preferences matches mutation profiles of natural homologues

The application of RECON MSD is not only optimized to engineer a stable, flexible protein, which is also readily accomplished by methods such as consensus sequence design (6). Rather, RECON MSD explores the sequence space consistent with a protein’s flexibility. Thus, we hypothesized, that the sequence space sampled by RECON MSD has similarity to the sequence space sampled in evolution. However, RECON MSD might explore additional sequences which have not yet been explored by natural selection. To answer this question, we compared calmodulin sequence profiles predicted by RECON MSD and SSD to naturally selected calmodulin sequence variation within a curated dataset of calmodulin representative of evolution across all eukaryotes (7). We also compared the mutation profiles of influenza virus HA2 predicted by either design with mutation profiles obtained from the NCBI Influenza Virus Resource database (IVR) (8). Given that the HA2 structural models used for this benchmark are of the influenza A H3N2 subtype (9-12), we included all influenza virus type A HA2 sequences deposited in the IVR to generate an HA2 profile. Briefly, in each case, we performed a multiple sequence alignment of all sequences within each database to generate sequence profiles. From each profile, we measured the root mean square deviation of mutation frequencies to corresponding mutation frequencies predicted by RECON MSD or SSD. For a complete description of the generation of sequence profiles, please consult the Methods section.

The calmodulin and influenza virus type A HA2 mutation frequencies had a root mean standard deviation of 0.473 and 0.580 with respect to RECON MSD profiles and 0.632 and 0.799 to SSD profiles, respectively (Fig 5). Although RECON MSD profiles were more likely to match the mutation tolerances observed within either multiple sequence alignment, the improvement was not uniform for all residues. In general, RECON MSD was more likely to predict matching mutation profiles for residues that undergo local conformational rearrangements, For calmodulin, RECON MSD was more likely to improve the prediction of mutation tolerances within the calmodulin EF-hand at conserved motif positions 3, 5, 9, and 12 (13). Even though neither ligands nor water molecules were included during protein design, sequence profiles for positions 9 and 12, which are known to provide a bridged water or direct binding to Ca^2+^, respectively, were similar. Within HA2 RECON MSD profiles, residues, charged residues within the B loop, which rearranges into an alpha helix in the post-fusion conformation, and the S5 loop region, which stabilizes the rearranged B loop alpha helix (14), were more likely to have similar predicted mutational tolerances as observed in influenza type A mutation profiles. However, in some cases, RECON MSD residue profiles either failed to improve or even worsened in predicting mutation profile similarity to profiles obtained from multiple sequence alignments as compared to SSD. This was particularly true for positions with small, non-polar residues that maintained contacts within the loosely packed interior within the HA2 trimer, including S93, V100, S113, and I149. Additionally, RECON MSD poorly predicted the conservation of charged residues whose side chains faced the protein interior in at least one conformation of either calmodulin or HA2 trimer, including calmodulin residues Q47, R84, D53, D90, Q132, and D126, and HA2 residue H106.

Because we used only H3N2 backbones to predict HA2 mutation profiles, we subdivided the HA2 profile obtained from all influenza type A multiple sequence alignment into different groups and subtypes including H1 and H2 from group 1 and H3, H4, H7, and H3N2 from group 2 to compare design profile similarity to subtype-specific mutation tolerances (Fig 6, S4 Fig). Separation of HA2 sequences by subtype revealed a divergence in similarity according to related subtypes, with RECON MSD sampling mutation profiles much closer to subtypes within the H3 clade, including H3 and H4. Even so, a Levene’s test for equality of variances (15) comparing mutation frequency variance within the H3N2 profile generated by RECON MSD to any influenza subtype A HA2 IVR profile indicated no significant difference between mutation frequency variances within the RECON MSD H3N2 profile and any HA2 IVR subtype profile. This suggests that RECON MSD samples similar mutational tolerances found across naturally selected influenza subtype A mutation tolerances, despite the diverging similarity to group 1 profiles. Given the high sequence profile similarity between RECON MSD and IVR H3 clade profiles, we conclude that RECON MSD can be used to predict possible sequence variation of closely related homologues from a single sequence. This might be particular useful in the case of predicting common mutations that arise due to genetic drift or reassortment, given that the HA2 profile modeled by RECON MSD was not significantly different from multiple HA2 subtype profiles. In cases where RECON MSD substantially deviates from observed mutation frequencies, particularly from the consensus sequence, such mutations warrant further experimental investigation to examine whether these stabilizing mutations within the H3 clade are artifacts of the Rosetta energy scoring function and/or RECON algorithm sampling, or that they indeed are evolutionary unexplored stabilizing mutations.

*The following citations were added, corresponding to the numbers listed below.*

6. Sternke M, Tripp KW, Barrick D. Consensus sequence design as a general strategy to create hyperstable, biologically active proteins. Proc Natl Acad Sci U S A. 2019;116(23):11275-84.

7. Halling DB, Liebeskind BJ, Hall AW, Aldrich RW. Conserved properties of individual Ca2+-binding sites in calmodulin. Proc Natl Acad Sci U S A. 2016;113(9):E1216-25.

8. Bao Y, Bolotov P, Dernovoy D, Kiryutin B, Zaslavsky L, Tatusova T, et al. The influenza virus resource at the National Center for Biotechnology Information. J Virol. 2008;82(2):596-601.

9. Weis WI, Brunger AT, Skehel JJ, Wiley DC. Refinement of the influenza virus hemagglutinin by simulated annealing. J Mol Biol. 1990;212(4):737-61.

10. Russell RJ, Kerry PS, Stevens DJ, Steinhauer DA, Martin SR, Gamblin SJ, et al. Structure of influenza hemagglutinin in complex with an inhibitor of membrane fusion. Proc Natl Acad Sci U S A. 2008;105(46):17736-41.

11. Chen J, Skehel JJ, Wiley DC. N- and C-terminal residues combine in the fusion-pH influenza hemagglutinin HA(2) subunit to form an N cap that terminates the triple-stranded coiled coil. Proc Natl Acad Sci U S A. 1999;96(16):8967-72.

12. Bullough PA, Hughson FM, Skehel JJ, Wiley DC. Structure of influenza haemagglutinin at the pH of membrane fusion. Nature. 1994;371(6492):37-43.

13. Zhou Y, Frey TK, Yang JJ. Viral calciomics: interplays between Ca2+ and virus. Cell Calcium. 2009;46(1):1-17.

14. Ekiert DC, Friesen RH, Bhabha G, Kwaks T, Jongeneelen M, Yu W, et al. A highly conserved neutralizing epitope on group 2 influenza A viruses. Science. 2011;333(6044):843-50.

Fig 5. Comparison of mutation profiles predicted by protein design to mutation profiles observed within calmodulin and influenza type A HA2 multiple sequence alignments. (A) Comparison of root mean square deviation of mutation frequencies derived from calmodulin natural homologues to mutation profiles predicted by RECON MSD or SSD. Calmodulin natural homologue mutation preferences were derived from the multiple sequence alignment of calmodulin homologues. The root mean square deviation (RMSD) here represents the mean standard deviation of an individual residue’s mutation profile, consisting as the mean sum of squared differences of all twenty amino acid frequencies as determined by the multiple sequence alignment of calmodulin homologue sequences in relation to either RECON MSD or SSD residue profile. (B) Residue profile standard deviations between calmodulin multiple sequence alignment profiles and design profiles mapped onto the unbound conformation of calmodulin (PDB ID 1CLL). Here, RMSD represents the mean sum of squared differences of all twenty amino acid frequencies of each residue between homologue and design profiles. Residues whose sequence profiles were predicted to have identical mutation profiles as that within the corresponding position with the multiple sequence alignment are colored in white. The greater the dissimilarity between the homologue mutation profile and design profile, the greater the saturation in red, with complete saturation indicating an RMSD of 1.0. Residues within all four of the conserved EF-hand motifs are labeled, with the bidentate ligand at position 12 critical for Ca^2+^ binding labeled in boldface. (C) Comparison of root mean square deviation of mutation frequencies derived from influenza type A sequence alignments to mutation profiles predicted by RECON MSD or SSD. RMSD is calculated in a similar fashion as in Panel A. (D) Residue profile standard deviations between HA2 multiple sequence alignment profiles and design profiles mapped onto the pre-fusion conformation of the HA2 trimer (PDB ID 2HMG). RMSD is calculated and labeled the same as in Panel B, but only one HA2 monomer is labeled with RMSD values of the influenza A IVR residue profiles in relation to RECON MSD or SSD profiles. The N- and C-terminal residues of loop regions that undergo large local conformational rearrangements in the post-fusion form are labeled. This includes the B loop that rearranges into an alpha helix and the S5 domain, which stabilizes the alpha helical form of the B loop. Residues within the CR8020 broadly neutralizing epitope (14), including N146 and E150, are also labeled.

*The following citation was added:*

14. Ekiert DC, Friesen RH, Bhabha G, Kwaks T, Jongeneelen M, Yu W, et al. A highly conserved neutralizing epitope on group 2 influenza A viruses. Science. 2011;333(6044):843-50.

Fig 6. Root mean square deviations of residue mutation frequencies of influenza A subtypes and HA2 profiles predicted by RECON MSD and SSD. (A) Dendrogram of root mean square deviations (RMSD) of influenza A subtype HA2 profiles sorted by pairwise RMSD. The mutation frequencies derived from the multiple sequence alignment profile of each influenza A subtype was compared to all other subtypes by calculating the mean standard deviation of each aligned position’s mean sum of squared differences of all twenty amino acid frequencies with respect to each other subtype profile. Pairwise RMSD values were sorted to form clades, with the height along the y axis indicating the pairwise RMSD between each clade. (B) RMSD of each IVR subtype multiple sequence alignment (MSA) profile with respect to RECON MSD and SSD. The x axis represents each IVR subtype profile sorted as in Panel A. The y axis represents the RMSD, calculated in the same fashion as in Panel A, of each subtype profile in relation to either RECON MSD or SSD.

S4 Fig. Root mean square deviation of residue mutation preferences between influenza A subtype multiple sequence alignments and their RECON MSD and SSD profiles. Each IVR subtype mutation profile was generated by multiple sequence alignment of HA2 sequences within the IVR database, subdivided by HA subtype including H1, H2, H3, H4, and H7. Because the designed sequence used only an H3N2 HA2 backbone, the H3N2 subtype was included in addition to H3. Only positions that align to the native sequence used for design were included within the profile. HA2 subsequences are separated and ordered by similarity to H3N2, from highest similarity on the top. The x axis each aligned position of the HA2 sequence, corresponding to the H3N2 residue numbering of PDB ID 2HMG, chain F. The y axis is the root mean square deviation (RMSD) of each residue’s subtype-specific profile within the multiple sequence alignment with respect to RECON MSD, on the left, and to SSD on the right.

*The following was revised the* ***Discussion*** *section:*

With that being said, we used RECON MSD to demonstrate that sequence conservation and mutation preferences of a single sequence can be approximated using the evaluation of local rearrangements in residue environment, provided that this one sequence folds into select, multiple conformations. In Fig 3, we showed that the estimated sequence conservation of RECON MSD designs differs by roughly 5% from the sequence conservation observed in PSI-BLAST profiles, with RECON MSD being more conservative. More specifically, we demonstrated that RECON MSD samples a very similar sequence space for hemagglutinin (HA2) compared to what has been observed in H3 clade influenza subtypes (Fig 6). Being able to predict the tolerated sequence space for viral antigens such as influenza HA has possible applications for antiviral drug design. It would have been preferred to compare the design profiles to deep sequencing data, as the represented mutation frequencies within the IVR database likely underestimate rare mutations. However, given the correspondence of the RECON MSD predicted HA2 sequence profiles and IVR subtype-specific sequence profiles, it stands to reason that RECON MSD can serve an *in silico* approximation for costly deep sequencing, or at least serves as an initial screening for potential, more frequently observed mutations of drug targets.

*The following was added to the* ***Methods*** *section:*

### Preparation of calmodulin and influenza type A HA2 mutation profilese

The entire influenza type A HA sequences were obtained from the IVR using the following parameters – type: A, Host: any, Country/Region: any, Protein: HA, Subtype H: any, Subtype N: any. Subdivision of the entire influenza type A HA sequences were obtained by changing the parameter Subtype H to 1, 2, 3, 4, or 7 with Subtype N as any, or for the H3N2 subtype, Subtype H: 3 and Subtype N:2. The number sequences obtained from each query are listed in Table 2. Sequences within the calmodulin dataset containing non-redundant calmodulin sequences across eukaryotes obtained from the Supplementary Material in (7) or influenza type A full-length HA sequences obtained from the Influenza Virus Resource (8) were aligned using a locally installed Clustal Omega version 1.2.4 (16, 17). Given the limited number of sequence gaps and high sequence conservation, we believe the multiple sequence alignments performed on either dataset were accurate. The frequency of each amino acid type at each aligned position was determined using WebLogo (18) using the following parameters – sequence-type: protein, format: logodata, composition: none.

### Table 2. Number of sequences within each influenza type A HA sequence dataset

| Influenza Type A subtype | Number of Sequences in IVR |
| --- | --- |
| All | 73943 |
| H3N2 | 29698 |
| H3 | 32645 |
| H4 | 1823 |
| H7 | 2424 |
| H1 | 23688 |
| H2 | 608 |

*The following references correspond with the citations used above:*

25. Bao Y, Bolotov P, Dernovoy D, Kiryutin B, Zaslavsky L, Tatusova T, et al. The influenza virus resource at the National Center for Biotechnology Information. J Virol. 2008;82(2):596-601.

26. Halling DB, Liebeskind BJ, Hall AW, Aldrich RW. Conserved properties of individual Ca2+-binding sites in calmodulin. Proc Natl Acad Sci U S A. 2016;113(9):E1216-25

50. Crooks GE, Hon G, Chandonia JM, Brenner SE. WebLogo: a sequence logo generator. Genome research. 2004;14(6):1188-90.

55. Sievers F, Wilm A, Dineen D, Gibson TJ, Karplus K, Li W, et al. Fast, scalable generation of high-quality protein multiple sequence alignments using Clustal Omega. Molecular systems biology. 2011;7:539.

56. Sievers F, Higgins DG. Clustal Omega for making accurate alignments of many protein sequences. Protein science : a publication of the Protein Society. 2018;27(1):135-45

**Minor comments:**
**In parts, the paper lacks clarity and some of the suggestions below are meant to clarify:**

*We thank the reviewer for thoroughly going through our manuscript to identify points which have not been made clear. We have added or changed the manuscript in anticipation that our more thorough explanation resolves any miscommunications or unclarities, highlighted in yellow.*

**1. The abstract states an increase of 30-40% in sequence conservation, but it is not clear how this is measured at this point. Either rephrase more accurately or eliminate the details here. Also, "RECON designs resembled the evolutionary sequence space of functional proteins" is both vague and as stated in Major comment above, largely to be expected for a conservative method.**

*The following was edited in the* ***Abstract****:*

RECON MSD across all conformations increased sequence conservation when compared to single-state designs in all cases. More importantly, the sequence space sampled by RECON MSD resembled the evolutionary sequence space of flexible proteins, particularly when confined to predicting the mutational preferences of limited common ancestral descent, such as in the case of influenza type A hemagglutinin. Additionally, we found that sequence positions which require substantial changes in their local environment across an ensemble of conformations are more likely to be conserved. These increased conservation rates are better captured by RECON MSD over multiple conformations and thus multiple local residue environments during design.

**2. I recommend to eliminate the first sentence of the introduction. It's far too broad for the current paper.**

*We agree with the reviewer, and we removed the first sentence and found it unnecessary to add a replacement first sentence.*

**3. The letter to the editor mentions that the method may lead to anticipating escape mutations in pathogens. I was intrigued by this suggestion but could find no mention for it in the paper. If the authors think this is realistic, they should explain in the Introduction/Discussion or provide some suggestive data.**

*We appreciate that the reviewer for pointing out this oversight, and we believe we addressed this concern while addressing his/her major concern.*

**4. line 64: "we hypothesize": it's trivial to assume that functionally essential flexibility limits sequence space. The word hypothesize is an overstatement. In particular, the authors cite other papers that already made this claim***.*

*The following was edited in the* ***Results*** *section, paragraph 1:*

It is our aim to demonstrate that simultaneous evaluation of sequence space across an ensemble of conformations improves the correspondence of the designed sequences to an evolutionary sequence profile by considering the constraints that local and global protein flexibility impose on amino acid identity and rotamer placement. For this benchmark, we perform RECON MSD and compare the designed profiles to SSD and evolutionary profiles.

**5. As the introduction is currently written, to understand the current contribution properly, the reader needs to have a fairly in-depth acquaintance with RECON and its predecessors, particularly the one from the Kortemme lab. I suggest to explain more deeply both of these methods to provide the necessary detail.**

*We agree with the reviewer that our cursory explanation of previously used methods failed to convey how RECON MSD is different from previously used methods that are suitable for sampling conformational plasticity of larger ensembles. We have expanded our explanation of these methods in the* ***Introduction*** *to explain their strengths and contributions to using protein ensemble design as a means to sample natural homologues’ sequence diversity.*

Humphris-Narayanan and colleagues demonstrated that prediction of mutation preferences of HIV-1 protease and HIV-1 reverse transcriptase was improved up to 25% when structural ensembles were included during protein design, as opposed to design of a single conformation (19, 20). The structural ensembles used for this approach were generated by reverting all structural side chains to a consensus sequence and employing Rosetta Backrub to introduce small local rotation about the C_α_-C_α_ axis of each of three-residue segments while maintaining ideal bond length, angle, and the starting χ1 angle (21, 22) at sites distributed throughout the protein known to acquire mutations. Next, protein design was performed on each backbone within the ensemble to select for mutations sequence that contributed to increased protein fold, dimer, and peptide stability, which was calculated as the lowest weighted sum of energy scores. They found that the substitution frequency of the consensus sequence, or profile, of the backbone ensemble better corresponded to the mutation frequencies observed within the Stanford HIV-1 Database (23) than the substitution frequencies obtained from design of an individual conformation. Additionally, they showed that the sequence profiles acquired with their Backrub ensembles were similar to the sequence profiles attained using an ensemble of experimentally-derived structural models. With such results, this approach succeeded in showing that representation of conformational plasticity during protein design better mimicked the mutational tolerances of HIV-1 protease and reverse transcriptase. The authors attribute this finding to the requirement of small backbone changes to accommodate mutations from the starting sequence.

For certain proteins, sub-Angstrom perturbations of the peptide backbone are insufficient to represent the conformational space consistent with their function. In the case of ubiquitin, Friedland and colleagues also used Rosetta Backrub to generate ubiquitin ensembles, but unlike in the previously mentioned method, they randomly inserted local rotations about the C_α_-C_β_ axis of two to twelve residues to diversify the conformational space of the generated ensembles. They then culled any generated models which disagreed with nuclear magnetic resonance (NMR) residual dipolar coupling constraints (RDCs), thus generating ubiquitin ensembles more similar to native-state solution dynamics (24). Using these RDC-constrained ensembles for design, they demonstrated that the mutation profiles obtained from the collection of individually-designed poses were more consistent with sequences within the ubiquitin family. In combination with the aforementioned study, these approaches demonstrated that a requirement for protein flexibility of a native-state ensemble substantially dictates the sequence space available for evolution.

*For these revisions, we added the following citations corresponding to the numbering used in the manuscript:*

3. Smith CA, Kortemme T. Backrub-like backbone simulation recapitulates natural protein conformational variability and improves mutant side-chain prediction. J Mol Biol. 2008;380(4):742-56.

4. Smith CA, Kortemme T. Predicting the tolerated sequences for proteins and protein interfaces using RosettaBackrub flexible backbone design. PloS one. 2011;6(7):e20451.

5. Rhee SY, Gonzales MJ, Kantor R, Betts BJ, Ravela J, Shafer RW. Human immunodeficiency virus reverse transcriptase and protease sequence database. Nucleic Acids Res. 2003;31(1):298-303.

**6. Line 100: "select the lowest-energy sequence": ill-defined. MSD methods select a sequence that finds a compromise among all states, not the lowest-energy sequence.**

*We adjusted the language used in the* ***Introduction*** *section:*

Using a pre-defined scoring function, positive-state MSD approaches rank the stability of a sequence as the average stability when threaded over each state across the ensemble (25). For most MSD approaches, replacement of the starting, or native, sequence occurs only if the energy of the mutant improves with respect to the starting sequences.

*For these revisions, we added the following citation corresponding to the numbering used in the manuscript:*

7. Davey JA, Chica RA. Multistate approaches in computational protein design. Protein science : a publication of the Protein Society. 2012;21(9):1241-52.

**7. Lines 245-246 explain the sequence-recovery benchmark. This is critical for understanding the results but I couldn't make sense of this explanation and I therefore only have a vague idea.**

*We altered the* ***Results*** *subsection “RECON MSD samples sequence profiles that are more similar to evolutionary observed sequence profiles when compared to SSD” to clarify the description of our benchmark.*

We first examined the correspondence of native sequence recovery determined by MSD versus SSD designed sequences to conservation rates within natural homologues. To accomplish this goal, we performed either RECON MSD or SSD on each set of protein conformations, allowing for the substitution of the native residue to all twenty amino acids and ignoring the presence of any disulfide bonds present in the native model. Designed sequence profiles of each conformation were generated using ten designed sequences, which were selected from either the ten lowest-scoring designed ensembles, as in the case of RECON MSD, or conformations for SSD. The mean total score of all conformations designed during the same RECON MSD run was used to sort and select the ten lowest-scoring designed ensembles. From the ten mean lowest-scoring ensembles, the set of ten models of each conformation was used for analysis. For SSD, each conformation was designed independently, and therefore the ten lowest-scoring models of each conformation were used for analysis. Therefore, the distinction between RECON MSD and SSD selected models rested in whether or not each of the ten selected models of each conformation where evaluated for design collectively as an ensemble or not. To compare the designed sequence profiles to that of the sequence diversity in natural homologues, the native sequence was used as the PSI-BLAST query sequence to generate PSI-BLAST profiles for each protein. Native sequence recovery was calculated as the mean percentage of conservation of the starting, or native, sequence for all designed positions, and for consistency, we term the percentage of the query sequence used to generate each PSI-BLAST profile as the percent native sequence recovery.

**8. similarly in lines 257-261**

*We adjusted the* ***Results*** *section “RECON MSD samples sequence profiles that are more similar to evolutionary observed sequence profiles when compared to SSD” to clarify the method we used to compare sequence profiles.*

Therefore, we calculated an average total deviation score of each observed position-specific mutation profile to the corresponding PSI-BLAST profile of each protein (Fig 3B and Fig S1), which depicts the root mean square deviation of all aligned positions’ profiles between a natural homologues’ mutational preferences and those predicted by design. We found that in seven out of eight cases, a RECON MSD mutation profile resembled its corresponding PSI-BLAST profile more closely than the SSD mutation profile, as the root mean square deviation was lower for comparisons between PSI-BLAST profiles and RECON MSD profiles than that of between SSD profiles.

**Reviewer #2:** **The main objective of protein design is to determine a favorable amino acid sequence for a given 3D structure. But this problem becomes quickly intractable due to the large amino acid sequence space from which a suitable sequence must be identified. Therefore, state-of-the-art methods make use of heuristic algorithms to explore the sequence space. Moreover, it is evident that these methods have demonstrated success by designing proteins de novo for developing therapies. In most of these studies, the authors have sought an amino acid sequence for the most energetically stable protein structure or a single state conformation. However, these methods are not necessarily helpful for designing a class of (flexible) proteins that exist in multiple conformational states or an ensemble. The complexity of such design problem increases significantly even for small flexible proteins. RECON (REstrained CONvergence) is an efficient method that can design sequences for an ensemble of conformations of large flexible proteins as shown in studies published in 2015 and 2019.

In this study, Sauer and colleagues utilize RECON to perform multi-state design (MSD) of large flexible proteins and demonstrate that the sequence profiles predicted favor conformational change. In particular, the designed sequences resemble evolutionary sequence profiles and tolerate mutations across all the conformations of an ensemble. Here, the authors compare sequences designed for a benchmark set of eight proteins, using RECON MSD with (i) sequences designed for each single conformation (single-state design or SSD) of an ensemble, and (ii) PSI-BLAST sequence profiles. The success of RECON MSD is obvious from its native sequence recovery which is 88% compared to 49% recovery from SSD or 82% from PSI-BLAST on average. Through alternative tests, authors show that RECON MSD doesn’t favor mutations as much as SSD, but the preferred mutations are close to those observed in evolution. While sequences sampled from RECON MSD are not as stable energetically as SSD sequences, they recognize mutations tolerated within the native structures. Here, the authors also establish a new metric called contact proximity deviation, that can be used to quantify changes in contact maps that capture the magnitude of local and global conformational flexibility across a structural ensemble. Later, they show that sequence conservation in the flexible regions depends heavily on the contact proximity deviation score (Fig 5). Through RECON MSD and contact proximity deviation score, the authors further demonstrate that they are able to predict sequences that are stable within the context of an ensemble but not necessarily for individual conformations when considered separately, thereby taking into account changes in local side-chain environment in the ensemble. The authors also present current limitations of the RECON MSD such as sampling bias from Rosetta and overestimation of sequence conservation in the hinge regions. This method is efficient given that it took up to 36 hours for proteins up to 1300 residues.

In summary, the current manuscript by Sauer et al demonstrates that RECON MSD can be utilized to design evolutionary sequence profiles of large flexible proteins. This can be very useful in understanding tolerated mutations in native proteins that are not yet revealed by evolution. Furthermore, the authors introduce a new metric that measures local conformational flexibility that occurs due to large structural changes. In general, the manuscript is very well-written and presents data in a logical and sound manner.**

*We thank this reviewer for a very positive review of our manuscript.*

**Minor Comments:**

**1. In the section “RECON MSD samples sequence profiles that are more similar to evolutionary observed sequence profiles when compared to SSD”, it is demonstrated that SSD favors mutation over RECON MSD. But, from section “Regions critical for conformational plasticity are energetically frustrated”, it seems like the opposite is true. These seem like they are contradicting one another. Can you please clarify?**

*We would like to thank the reviewer for pointing out this miscommunication. First, we felt it necessary to rename the section, since we are not specifically dissecting regions that are or are not critical for conformational plasticity. Second, besides addressing the other reviewer’s concerns in the section “RECON MSD samples sequence profiles that are more similar to evolutionary observed sequence profiles when compared to SSD”, we felt that this section did not require additional revisions. However, we agree that the retitled section, shown below, should be rewritten to avoid any contradictions, which we have highlighted in yellow.*

## Sequences suitable for conformational plasticity are energetically frustrated

The encouraged sequence convergence employed by the RECON MSD algorithm identifies amino acid sequences that have the lowest total energy across all states (26). To examine the energetic impact of requiring a single amino acid sequence to adopt multiple states, we use a similar energy score term described previously as the sum total energy score normalized by the number of designed positions (see Methods). For RECON MSD designs, this approach would include lowest mean energy score of the designed ensemble, whereas the SSD energy score would include the lowest energy scores for each state. In all eight cases, RECON MSD selects sequences with a significantly higher energy score than SSD with a paired student’s t-test (27), with $p<1\times{10}^{-4}$ (Fig 7). We also compared the design energy scores to the ten lowest-energy relaxed structures, which only included the native sequences, and found that RECON MSD samples lower energy sequences relative the relaxed native structures. Given that RECON MSD conserves, on average, 88% of native sequences, the few mutations RECON MSD introduces to the native sequence are sufficient to sample a lower energy sequence space than the native sequence. In comparison, SSD sequences are the most stable as SSD replaces the native sequence at a much higher frequency, since SSD optimizes the sequence space for each conformation and can identify much lower energy sequences tolerable for each individual conformation. Therefore, given that RECON MSD is constrained to identify sequences that are suitable to adopt multiple conformations and that the sequence space identified by RECON MSD is, on average, higher in total energy than the sequence space identified by SSD, it is implied that the sequence space available for proteins that populate multiple energy minima is more likely to be energetically frustrated, or at least not as energetically stable, than the sequence space available for a protein which populates a single energy minimum. **2. References were not found in lines: 408, 623, 633, 647.**

*We thank the reviewer for identifying missing references. We have added the following references to the corresponding missing references as listed above:*

- Kendall MG. Rank Correlation Methods. 2 ed. Griffin C, editor: The University of California; 1948. 160 p.
- Weiss NA. A Course in Probability: Addison-Wesley; 2005.
- Yampolsky LY, Stoltzfus A. The exchangeability of amino acids in proteins. Genetics. 2005;170(4):1459-72
- Kufareva I, Abagyan R. Methods of protein structure comparison. Methods in molecular biology. 2012;857:231-57.

**3. Figure S4 is not referenced anywhere (or maybe it is one of the references that is missing as pointed out in my previous comment). Can you please elaborate on this? I think this should further clarify why RECON MSD sequence conservation is dependent on both RMSDda and contact map deviation shown in Figure 5.**

*We thank the reviewer for bringing this oversight to our attention, and we agree that Figure S5 (previously S4 Fig) was intended to address the relationship of RMSD_da_ and contact map deviation. We found it necessary to address your concern in the last section regarding the relationship of each conformational flexibility metric and energy score, so that we were able to address the contribution of energy score to native sequence recovery.*

There was no dependence of individual residue scores on RMSD_da_ for either design approach (Fig 8), which is surprising given that both native sequence recoveries for RECON MSD and SSD were strongly dependent on RMSD_da_. It should be noted that we found the metrices RMSD_da_ and contact proximity deviation were not independent variables, as we determined that contact proximity deviation is significantly, although not strongly, negatively correlated with RMSD_da_ (S7 Fig), meaning that residues with contact proximity deviation values close to or at zero were also more likely to have a higher RMSD_da_ values. In either design case, residues with high RMSD_da_ values tend to have lower scores, *i.e.* score favorably, suggesting that the native contacts formed at positions restricted in rearranging side chain proximities for one or all states were more likely to score favorably, or at least more favorably than non-native side chains, by the Rosetta scoring function (34). A favorable reference score would prevent the native residue in being redesigned with non-native residues, hence the correlation of high RMSD_da_ values and higher native sequence recovery. Even so, the $\tau_{\beta}$ correlation of RMSD_da_ and designed sequence energy was not significant in either design case, such that the degree of backbone flexibility does not directly influence a residue’s stability.

*The following citation was added in this revision:*

33. Leaver-Fay A, Tyka M, Lewis SM, Lange OF, Thompson J, Jacak R, et al. ROSETTA3: an object-oriented software suite for the simulation and design of macromolecules. Methods in enzymology. 2011;487:545-74.

*Below are the citations included in the response to the reviewers. Note that numbering will not correspond with the numbering used in the manuscript.*

1.	Zemla A. LGA: A method for finding 3D similarities in protein structures. Nucleic Acids Res. 2003;31(13):3370-4.

2.	Abagyan RA, Totrov MM. Contact area difference (CAD): a robust measure to evaluate accuracy of protein models. J Mol Biol. 1997;268(3):678-85.

3.	Marsden B, Abagyan R. SAD--a normalized structural alignment database: improving sequence-structure alignments. Bioinformatics. 2004;20(15):2333-44.

4.	Lange OF, Lakomek NA, Fares C, Schroder GF, Walter KF, Becker S, et al. Recognition dynamics up to microseconds revealed from an RDC-derived ubiquitin ensemble in solution. Science. 2008;320(5882):1471-5.

5.	O'Rourke KF, Gorman SD, Boehr DD. Biophysical and computational methods to analyze amino acid interaction networks in proteins. Comput Struct Biotechnol J. 2016;14:245-51.

6.	Sternke M, Tripp KW, Barrick D. Consensus sequence design as a general strategy to create hyperstable, biologically active proteins. Proc Natl Acad Sci U S A. 2019;116(23):11275-84.

7.	Halling DB, Liebeskind BJ, Hall AW, Aldrich RW. Conserved properties of individual Ca2+-binding sites in calmodulin. Proc Natl Acad Sci U S A. 2016;113(9):E1216-25.

8.	Bao Y, Bolotov P, Dernovoy D, Kiryutin B, Zaslavsky L, Tatusova T, et al. The influenza virus resource at the National Center for Biotechnology Information. J Virol. 2008;82(2):596-601.

9.	Weis WI, Brunger AT, Skehel JJ, Wiley DC. Refinement of the influenza virus hemagglutinin by simulated annealing. J Mol Biol. 1990;212(4):737-61.

10.	Russell RJ, Kerry PS, Stevens DJ, Steinhauer DA, Martin SR, Gamblin SJ, et al. Structure of influenza hemagglutinin in complex with an inhibitor of membrane fusion. Proc Natl Acad Sci U S A. 2008;105(46):17736-41.

11.	Chen J, Skehel JJ, Wiley DC. N- and C-terminal residues combine in the fusion-pH influenza hemagglutinin HA(2) subunit to form an N cap that terminates the triple-stranded coiled coil. Proc Natl Acad Sci U S A. 1999;96(16):8967-72.

12.	Bullough PA, Hughson FM, Skehel JJ, Wiley DC. Structure of influenza haemagglutinin at the pH of membrane fusion. Nature. 1994;371(6492):37-43.

13.	Zhou Y, Frey TK, Yang JJ. Viral calciomics: interplays between Ca2+ and virus. Cell Calcium. 2009;46(1):1-17.

14.	Ekiert DC, Friesen RH, Bhabha G, Kwaks T, Jongeneelen M, Yu W, et al. A highly conserved neutralizing epitope on group 2 influenza A viruses. Science. 2011;333(6044):843-50.

15.	Levene H. Robust tests for equality of variances: Stanford University Press; 1960. 517 p.

16.	Sievers F, Wilm A, Dineen D, Gibson TJ, Karplus K, Li W, et al. Fast, scalable generation of high-quality protein multiple sequence alignments using Clustal Omega. Molecular systems biology. 2011;7:539.

17.	Sievers F, Higgins DG. Clustal Omega for making accurate alignments of many protein sequences. Protein science : a publication of the Protein Society. 2018;27(1):135-45.

18.	Crooks GE, Hon G, Chandonia JM, Brenner SE. WebLogo: a sequence logo generator. Genome research. 2004;14(6):1188-90.

19.	Humphris-Narayanan E, Akiva E, Varela R, S OC, Kortemme T. Prediction of mutational tolerance in HIV-1 protease and reverse transcriptase using flexible backbone protein design. PLoS Comput Biol. 2012;8(8):e1002639.

20.	Christian EA, Kahle KM, Mattia K, Puffer BA, Pfaff JM, Miller A, et al. Atomic-level functional model of dengue virus Envelope protein infectivity. Proc Natl Acad Sci U S A. 2013;110(46):18662-7.

21.	Smith CA, Kortemme T. Backrub-like backbone simulation recapitulates natural protein conformational variability and improves mutant side-chain prediction. J Mol Biol. 2008;380(4):742-56.

22.	Smith CA, Kortemme T. Predicting the tolerated sequences for proteins and protein interfaces using RosettaBackrub flexible backbone design. PloS one. 2011;6(7):e20451.

23.	Rhee SY, Gonzales MJ, Kantor R, Betts BJ, Ravela J, Shafer RW. Human immunodeficiency virus reverse transcriptase and protease sequence database. Nucleic Acids Res. 2003;31(1):298-303.

24.	Friedland GD, Lakomek NA, Griesinger C, Meiler J, Kortemme T. A correspondence between solution-state dynamics of an individual protein and the sequence and conformational diversity of its family. PLoS Comput Biol. 2009;5(5):e1000393.

25.	Davey JA, Chica RA. Multistate approaches in computational protein design. Protein science : a publication of the Protein Society. 2012;21(9):1241-52.

26.	Sevy AM, Jacobs TM, Crowe JE, Jr., Meiler J. Design of Protein Multi-specificity Using an Independent Sequence Search Reduces the Barrier to Low Energy Sequences. PLoS Comput Biol. 2015;11(7):e1004300.

27.	Student. The probable error of a mean. Biometrika. 1908;6(1):1-25.
